# Efficacy and safety of hypoxia-inducible factor-prolyl hydroxylase inhibitor treatment for anemia in chronic kidney disease: an umbrella review of meta-analyses

**DOI:** 10.3389/fphar.2023.1296702

**Published:** 2023-11-30

**Authors:** Song Ren, Xiaoxiu Yao, Yi Li, Ying Zhang, Chao Tong, Yunlin Feng

**Affiliations:** ^1^ Department of Nephrology, Sichuan Provincial People’s Hospital, University of Electronic Science and Technology of China, Chengdu, China; ^2^ Department of Health Management, Damian Honghe Community Health Service Center of Longquanyi District, Chengdu, China; ^3^ Department of Ophthalmology, Sichuan Provincial People’s Hospital, University of Electronic Science and Technology of China, Chengdu, China; ^4^ State Key Laboratory of Maternal and Fetal Medicine of Chongqing Municipality, The First Affiliated Hospital of Chongqing Medical University, Chongqing, China; ^5^ Renal Division, The George Institute for Global Health, University of New South Wales, Sydney, NSW, Australia

**Keywords:** hypoxia-inducible factor-prolyl hydroxylase inhibitor, anemia, chronic kidney disease, hemoglobulin, iron metabolism, safety, umbrella review

## Abstract

The objective was to provide a comprehensive summary of existing evidence on the efficacy and safety of hypoxia-inducible factor-prolyl hydroxylase inhibitors (HIF-PHIs) for the treatment of anemia in chronic kidney disease (CKD). A systematic search was conducted in the Medline, Embase, and Cochrane databases. Only meta-analyses that evaluated the efficacy and safety of HIF-PHI treatment for anemia in CKD were included. The efficacy outcomes included hemoglobin levels and iron metabolism indices, while the safety outcomes were assessed by examining adverse events. The qualities of methodologies and evidence were assessed using the AMSTAR 2 system and the NutriGrade tool, respectively. Fourteen meta-analyses, comprising 105 distinct comparisons, were included. The comparisons were backed by evidence of high, moderate, and low levels, distributed in approximately equal proportions. None of the studies were deemed to possess a high level of confidence. In both the overall and individual treatment groups of HIF-PHI, there was an increase in the levels of hemoglobin, transferrin, and transferrin saturation, while the levels of hepcidin and total iron binding capacity decreased. Serum ferritin exhibited a reduction to some extent, while serum iron did not show significant alterations following HIF-PHI treatments. There were no notable disparities in safety outcomes between the HIF-PHI and erythropoietin stimulating agents or placebo groups. This umbrella review suggests that HIF-PHI treatment can effectively increase hemoglobin levels in CKD patients and enhance iron metabolism by decreasing hepcidin levels and improving iron transport. The safety profiles of HIF-PHIs were generally comparable to those of ESA therapies or placebos.

## Introduction

The prevalence of anemia is high in chronic kidney disease (CKD) (Stauffer and Fan, 2014) and escalates as renal function declines (Li et al., 2016). The occurrence of anemia in CKD patients, even of a mild degree, is associated with the risks of all-cause mortality, cardiovascular comorbidities, and progressive decline of renal function (Locatelli et al., 2014; Minutolo et al., 2022). Transfusion-based treatment for renal anemia heightens the likelihood of allosensitization for subsequent transplantation. Therefore, the management of anemia has consistently been regarded as a crucial aspect of clinical care for CKD and has received concentrated scrutiny. Erythropoietin stimulating agent (ESA) therapy and iron supplementation have substantially revolutionized the treatment of renal anemia for nearly four decades.

Nevertheless, several intricate scenarios continue to pose challenges, such as hyporesponsiveness to ESA, reduced effectiveness of ESA in the presence of inflammation and/or iron deficiency or when aiming at high Hb targets, safety concerns about high-dose ESA, as well as risks of infection, cardiovascular-related adverse effects, infusion reactions, sensitization, and iron overload associated with intravenous iron supplementation (Fishbane and Besarab, 2007; Litton et al., 2013; Karaboyas et al., 2015). It is in this context that there has been significant interest and rapid progress in the development of hypoxia-inducible factor (HIF)-prolyl hydroxylase inhibitor (PHI) agents (Chen et al., 2019a; Chen et al., 2019b; Mima, 2021). Numerous HIF-PHI drugs have demonstrated efficacy in increasing Hb levels in renal anemia, as evidenced by randomized controlled trials (RCTs) (Chen et al., 2019a; Chen et al., 2019b; Fishbane et al., 2022; Gang et al., 2022; Hou et al., 2022; Singh et al., 2022) and meta-analyses (Li et al., 2021; Zhang et al., 2021; Fatima et al., 2022; Takkavatakarn et al., 2023; Zheng et al., 2023).

Despite the existence of multiple meta-analyses examining the impact of HIF-PHIs on anemia in CKD (Li et al., 2021; Zhang et al., 2021; Fatima et al., 2022; Chen et al., 2023; Takkavatakarn et al., 2023; Yang et al., 2023; Zheng et al., 2023), the number of included studies in these meta-analyses varied and there is a lack of comprehensive synthesis regarding the overall quality of evidence. Given that umbrella reviews prioritize the inclusion of evidence from systematic review and assess included meta-analysis in both methodology and quality aspects, thereby occupying the apex of the evidence pyramid for clinical decision-making (Aromataris et al., 2015), we have undertaken the first umbrella review to comprehensively consolidate the available evidence from meta-analyses on the effectiveness and safety of HIF-PHIs in the treatment of anemia in CKD, aiming to provide healthcare providers with the highest level of evidence relevant to this topic.

## Methods

### Data sources and literature search

A systematic search was performed according to the Preferred Reporting Items for Systematic Review and Meta-Analyses (PRISMA) statement (Liberati et al., 2009) for systematic reviews or meta-analyses from inception through 25 May, 2023 in MEDLINE via PubMed, EMBASE via Ovid, and Cochrane Database of Systematic Reviews without date limitations, using text words and medical subject headings relevant to “hypoxia-inducible factor-prolyl hydroxylase inhibitor” and “kidney disease” (see Supplementary Table S1). This umbrella review was prospectively registered in PROSPERO (CRD42023429560).

### Study selection

Systematic reviews and meta-analyses assessing the efficacy and safety of HIF-PHIs for anemia in CKD were considered eligible for this umbrella review. Two reviewers (SR and XY) independently conducted the study selection following a standardized approach. Titles and abstracts of all identified records from the literature research were carefully reviewed. Reference lists of articles that had been reviewed in full text were further manually screened for eligible studies that could have been missed during the literature search.

Studies were excluded if they were duplicates, systematic reviews without meta-analyses, narrative reviews, network meta-analyses without extractable information for comparison, meta-analyses of individual patient data, protocols, comments, or editorials. Studies that reported interventions other than HIF-PHIs for anemia in CKD or outcomes irrelevant to efficacy or safety were also excluded. Corresponding authors of abstracts with insufficient data were contacted via e-mail for relevant information; however, no reply was received. Any discrepancy was adjudicated by a third reviewer (YF).

For each outcome comparison, only one meta-analysis was chosen to avoid duplicates. If more than one meta-analysis had been identified for the index comparison, the one with the largest number of primary studies was preferred. If more than one meta-analysis had included the same number of primary studies, the one with the largest number of patients was considered for inclusion. If more than one meta-analysis met the above two criteria, the one that reported more detailed outcomes was selected.

### Outcomes

The outcomes in this umbrella review were divided into the efficacy and safety outcomes. The efficacy outcomes referred to the therapeutic effects of HIF-PHIs on hemoglobulin and iron metabolism indices, including hepcidin, transferrin, total iron binding capacity (TIBC), transferrin saturation (TSAT), serum iron, and ferritin. The safety outcomes included any adverse events (AEs), severe adverse events (SAEs), major adverse cardiovascular events (MACEs), or vascular events. All outcomes had to include summary estimates to allow the pooling analysis.

### Data extraction

Data from eligible studies were extracted by two reviewers (SR and XY) independently and compiled onto a shared sheet. Any disagreement was resolved by the third reviewer (YF). The following data were extracted from each included meta-analysis: authors, publication year, targeted population, number of primary studies, number of patients, details of the experimental interventions, details of the controlled treatment, studied outcomes, metrics of the meta-analysis, effect model used to generate the metrics, estimates and 95% confidence interval (CI) of the metrics, heterogeneity indices, and indices of publication bias.

### Quality assessment of methodologies and evidence

Two reviewers (SR and YF) assessed the quality of methodologies of the included studies using the AMSTAR 2 system (Shea et al., 2017). The AMSTAR 2 system has 16 items that comprehensively evaluate the quality of meta-analyses and rates the overall confidence of the review based on assessment results of possible critical flaws and noncritical weaknesses. Each question can be answered with “yes” or “no.” Seven items are considered critical domains and have key weights in the overall rating. The overall quality was rated as “high,” “moderate,” “low,” or “very low” confidence based on the combinations of answers to the critical and noncritical items.

The quality of evidence for each comparison was also evaluated by two reviewers (SR and YF) using the NutriGrade tool (Schwingshackl et al., 2016). Since all included meta-analyses reported the pooled results of RCTs, seven items in the NutriGrade tool were used, including risk of bias (0–3 points), precision (0–1 point), heterogeneity (0–1 point), directness (0–1 point), publication bias (0–1 point), funding bias (0–1 point), and study design (0–2 points). The resulting scores of each item were summed to generate an overall score. The grade of evidence was assigned as “high,” “moderate,” “low,” and “very low” when the overall score was ≥8, 6 to <8, 4 to <6, and <4, respectively.

### Data analysis

For each studied outcome, estimates of effects were categorized based on the comparisons of interventions. HIF-PHIs were classified into a general group of HIF-PHIs and each individual HIF-PHI drug as reported. Controlled treatments were classified into ESA therapy, placebo, and ESA therapy combined with placebo as reported. To obtain an overview of comparisons for each outcome, different types of effects, including the mean difference (MD), standardized mean difference (SMD), risk ratio (RR), and odds ratio (OR), were not differentiated but were compiled together. R version 4.0.3 (R Project for Statistical Computing) was used for data synthesis. *p* < 0.05 was considered statistically significant.

## Results

### Characteristics of the included meta-analyses

**FIGURE 1:**
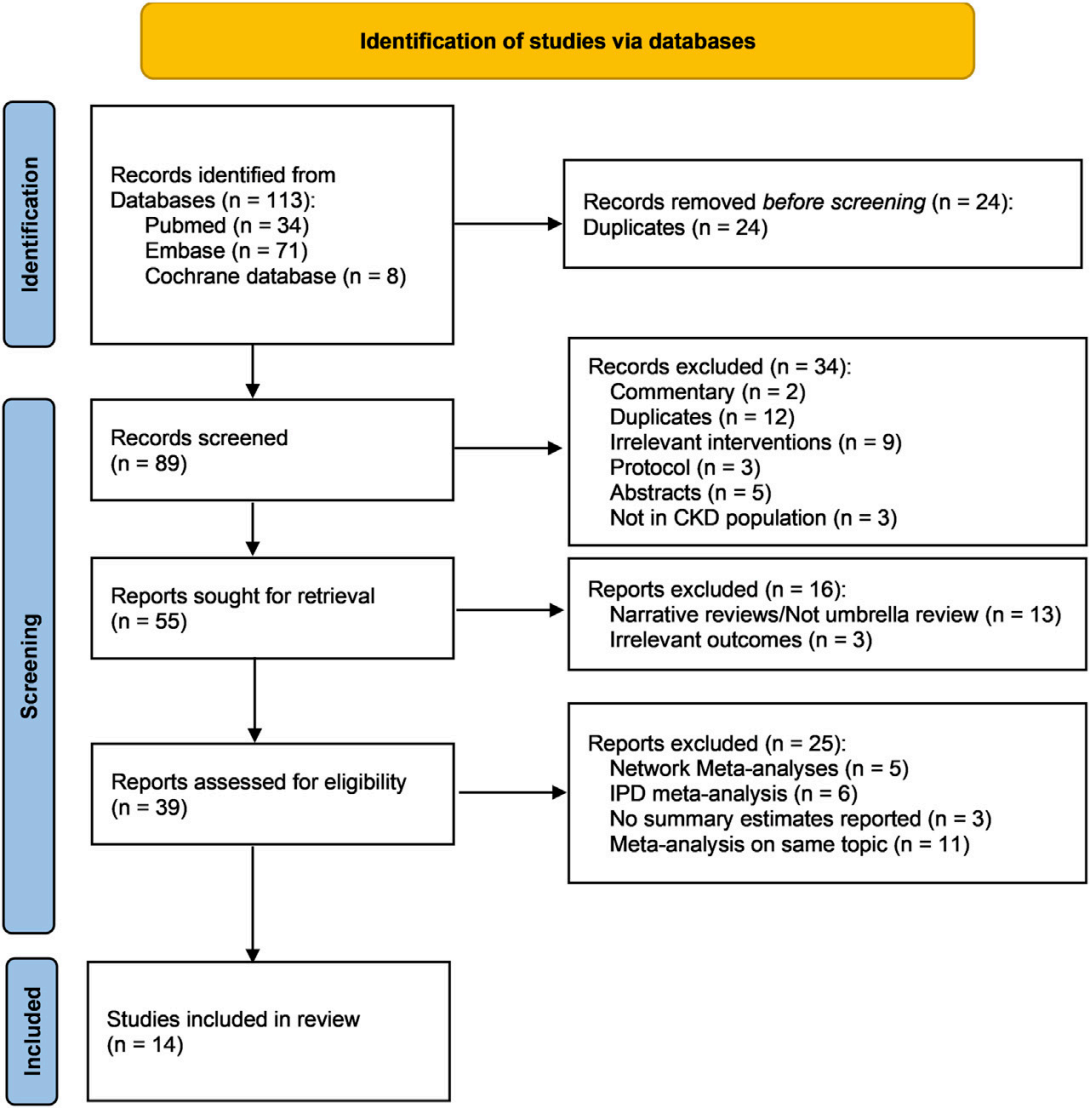
PRISMA flow chart of this umbrella review. Abbreviations: CKD, chronic kidney disease; IPD, individual patient data; n, number.

A total of 89 records were returned from the literature search after the removal of duplicates. After the title and abstract screening, 39 publications were kept for full text review, among which 25 were further excluded, leaving 14 systematic reviews for the umbrella review (Liu et al., 2020; Wang et al., 2020; Li et al., 2021; Liu et al., 2021; Qie et al., 2021; Xiong et al., 2021; Zhang et al., 2021; Zheng et al., 2021; Fatima et al., 2022; Lei et al., 2022; Wu et al., 2022; Mohamed et al., 2023; Takkavatakarn et al., 2023; Zheng et al., 2023) (Figure 1) (justification for the excluded articles after removing duplicates is presented in Supplementary Table S2).

The 14 eligible meta-analyses included a total of 105 unique comparisons for different outcomes, among which 41 (39.0%) comparisons were supported by a high level of evidence, 36 (34.3%) comparisons were supported by a moderate level of evidence, and 28 (26.7%) comparisons were supported by a low level of evidence (Supplementary Table S3). Quality assessment of methodologies using the AMSTAR 2 system indicated that more than half (8 of 14, 57.1%) of the included meta-analyses were rated as moderate confidence, 4 (28.6%) as low confidence, and 2 (14.3%) as very low confidence (Supplementary Table S4). None of the studies were rated as high confidence.

### Efficacy of HIF-PHIs on hemoglobulin

**FIGURE 2:**
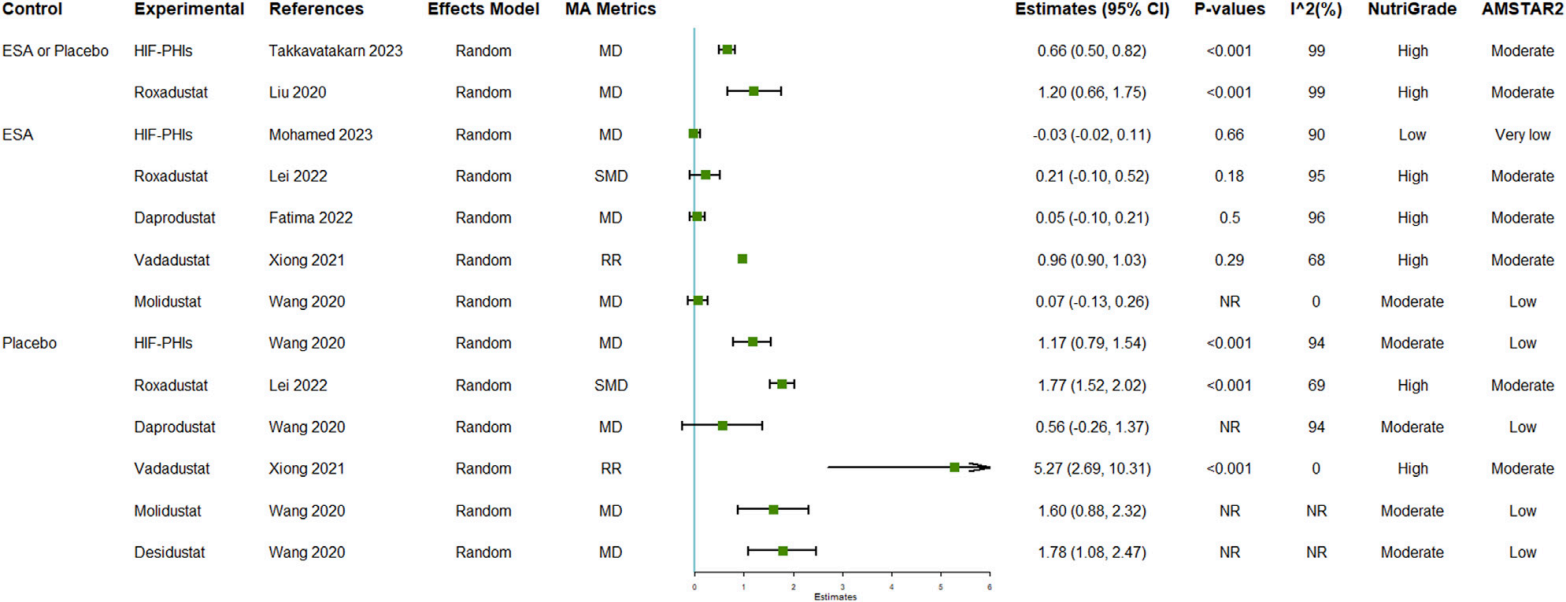
Efficacy of HIF-PHI treatment on hemoglobin compared with erythropoietin replacement or placebo. Abbreviations: EPO, erythropoietin; HIF-PHIs, hypoxia-inducible factor-prolyl hydroxylase inhibitors; MD, mean difference; NR, not reported; SMD, standardized mean differences.

The reported efficacy of HIF-PHIs on hemoglobulin contained 13 unique comparisons (Figure 2; Supplementary Table S5). Seven of the 13 comparisons were conducted in CKD patients, 5 were specifically conducted in non-dialysis-dependent CKD patients, and 1 was conducted in dialysis-dependent CKD patients. Although the pooled estimates of effect size revealed high heterogeneities, the overall results indicated that HIF-PHI treatments effectively elevated hemoglobulin levels by approximately 1 g/dL. The effects were more pronounced in the comparisons between HIF-PHIs and placebos than in the comparisons between HIF-PHIs and EPO for not only general but also individual HIF-PHI drugs.

### Efficacy of HIF-PHIs on iron metabolism

**FIGURE 3:**
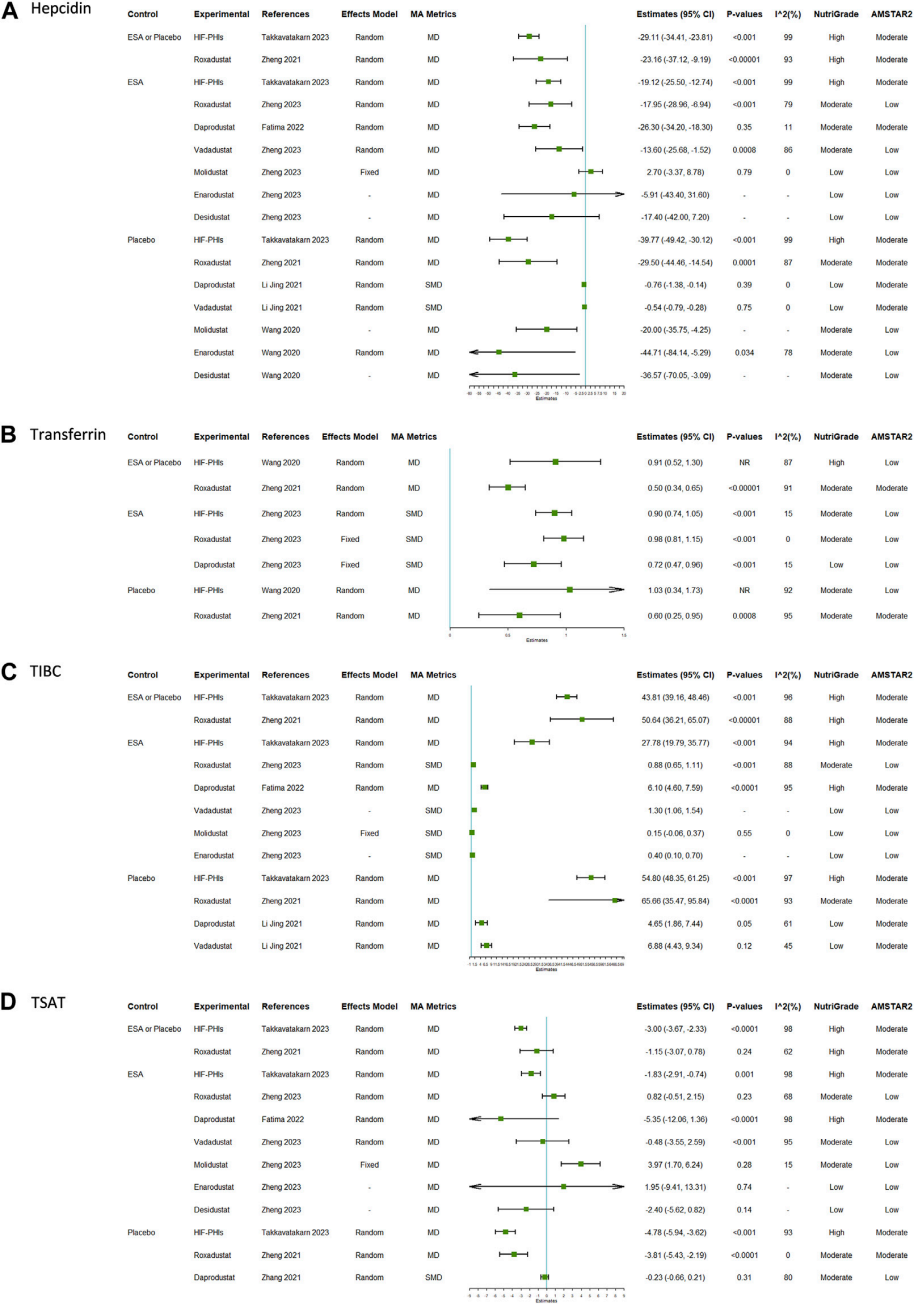
Efficacy of HIF-PHI treatment on iron metabolism indices compared with erythropoietin replacement or placebo: **(A)** hepcidin; **(B)** transferrin; **(C)** TIBC; **(D)** TSAT. Abbreviations: EPO, erythropoietin; HIF-PHIs, hypoxia-inducible factor-prolyl hydroxylase inhibitors; MD, mean difference; NR, not reported; SMD, standardized mean differences; TIBC, total iron binding capacity; TSAT, transferrin saturation.

The reported efficacy of HIF-PHIs on hepcidin contained 16 unique comparisons (Figure 3A; Supplementary Table S6). Eight of the 16 comparisons were conducted in general CKD patients, 5 were conducted in dialysis-dependent CKD patients, and 3 were conducted in non-dialysis dependent CKD patients. The overall results indicated that HIF-PHI treatments reduced the levels of hepcidin with greater amplitudes in comparison with placebos than in comparison with EPO. Molidustat was the only type of HIF-PHI that failed to reduce hepcidin; however, the difference was not significant.

There were 7, 12, and 12 unique comparisons for transferrin, TIBC, and TSAT, respectively. The pooled results indicated that treatment with HIF-PHIs consistently increased the levels of serum transferrin (Figure 3B; Supplementary Table S7) and TIBC (Figure 3C; Supplementary Table S8) but decreased the level of TSAT (Figure 3D; Supplementary Table S9). The proportion of effect size estimates with statistical significance for TSAT was lower than those for transferrin and TIBC.

There were 11 and 13 unique comparisons for serum iron and ferritin, respectively. A total of 63.6% (7 of 11) of the comparisons for serum iron demonstrated that HIF-PHI treatment did not alter serum iron (Supplementary Figure S1A; Supplementary Table S10). A total of 69.2% (9 of 13) of the comparisons for serum ferritin indicated that HIF-PHI treatment was able to decrease the levels of serum ferritin, with reductions to a greater extent in comparisons between HIF-PHIs and placebo than in comparisons between HIF-PHIs and ESA therapy (Supplementary Figure S1B; Supplementary Table S11).

### Safety of HIF-PHIs

**FIGURE 4:**
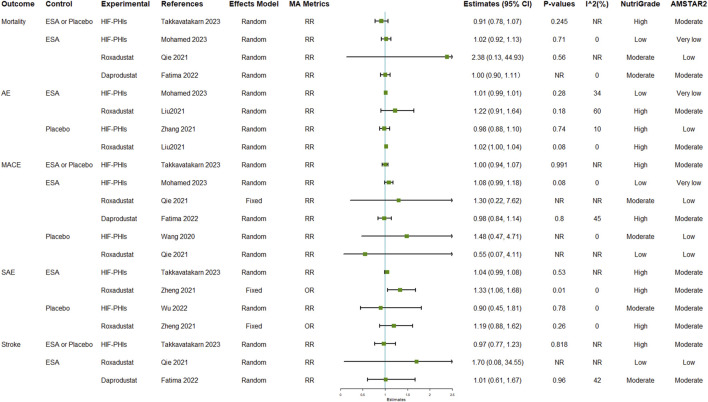
Safety outcomes of HIF-PHI treatment compared with erythropoietin replacement or placebo. Abbreviations: AE, adverse event; EPO, erythropoietin; HIF-PHIs, hypoxia-inducible factor-prolyl hydroxylase inhibitors; MACE, major adverse cardiovascular event; MD, mean difference; NR, not reported; OR, odds ratio; RR, risk ratio.

Mortality and MACE are the two safety outcomes that had been most reported. All comparisons with reported I^2^ results in this group of outcomes had I^2^ values of less than 75%, suggesting consistency of the pooled estimates of effect size. The pooled results indicated that all types of safety outcomes after HIF-PHI treatments, including mortality, all AEs, MACE, SAEs, and stroke, were generally not significantly different from those following either ESA or placebo, or combined (Figure 4; Supplementary Table S12). Notably, one meta-analysis indicated the incidence of SAE in the roxadustat group was significantly higher than that in the ESA group (OR: 1.33, 95% CI: 1.06–1.68, *p* = 0.01) (Zheng et al., 2021).

## Discussion

To the best of our knowledge, this is the first umbrella review to comprehensively synthesize existing evidence regarding the effectiveness and safety of HIF-PHIs for the management of renal anemia. Our findings demonstrate that HIF-PHI treatment consistently and effectively raises hemoglobulin levels in CKD patients. The levels of transferrin and transferrin saturation increased in the HIF-PHI treatment groups, alongside decreased levels of hepcidin and TIBC. While the level of serum ferritin exhibited a certain degree of reduction, serum iron levels did not significantly change in response to HIF-PHI treatments. The risks of any AE, SAE, MACE, and stroke generally did not significantly differ between the HIF-PHI groups and the control groups.

HIF-PHIs treat renal anemia via mechanisms that are different from those of ESA therapy and regulates erythropoiesis at multiple levels (Koury and Haase, 2015; Coyne et al., 2017). The results of this umbrella review provide strong evidence for the rapid correction of anemia with HIF-PHIs at the doses used in the trial, in line with the results of multiple RCTs (Holdstock et al., 2016; Akizawa et al., 2021; Yamamoto et al., 2021; Singh et al., 2022) and a series of meta-analyses (Li et al., 2021; Fatima et al., 2022; Takkavatakarn et al., 2023; Zheng et al., 2023). In this umbrella review, most of the meta-analysis that had reported pooled results on hemoglobulin were supported by a moderate or high level of evidence. In response to the rapid correction of anemia following HIF-PHI treatments, close monitor and examination are necessary for dosage adjustment. Additionally, the oral administration of HIF-PHIs might provide possible advantage in reducing the medical waste and work time of hemodialysis nurses.

Furthermore, HIF-PHIs have advantages in regulating iron metabolism in anemia. HIF-PHIs can enhance iron utilization by reducing the hepatic peptide hepcidin, which is a systemic iron-regulatory hormone regulating plasma iron concentrations, intestinal iron absorption, and tissue iron distribution (Ganz and Nemeth, 2012). Hepcidin limits iron availability, prevents the release of stored iron, and promotes internalization of the cellular iron exporter ferroprotein for degradation, thus negatively regulating iron utilization. Reportedly, the inflammatory environment in CKD induces the elevation of hepcidin, which may in turn require a higher dose of ESA therapy or result in hypo-responsiveness to ESA (Zheng et al., 2023). Our findings and existing evidence in the literature jointly proved that HIF-PHI treatment can facilitate the iron utilization by reducing the level of hepcidin.

HIF-PHIs may also improve iron metabolism by enhancing iron transportation. HIF-PHI treatment increases the expression of transferrin (Mima, 2021), which is a downstream effector of the HIF pathway, thus resulting in increased TIBC and subsequently decreased TSAT. The linkage changes among transferrin, TIBC, and TSAT in response to HIF-PHI treatment in renal anemia are the opposite to the results in iron deficiency anemia after iron supplementation, reflecting the different mechanisms in these two anemia scenarios. It should be noted that changes in parameters of iron metabolism may be partially due to differences in the erythropoietic activity of the agents. Additionally, attention should be paid to the notable differences of different HIF-PHI agents on the examined iron metabolism outcomes when interpretate the results.

Safety has been an area of active debate in the approval of HIF-PHIs, specifically the cardiovascular risk and increased VEGF following treatments (Mima, 2021). However, our findings in this study showed that HIF-PHIs are not different from ESA therapy or placebo regarding the overall as well as specific safety outcomes, and the low I^2^ values revealed the consistency of this result in the literature. Nevertheless, we should bear in mind that it has been only 4 years since roxadustat was approved in December, 2018 in China as the first HIF-PHI agent to treat renal anemia. More evidence from long-term and strict surveillance is warranted for a comprehensive evaluation of the safety profiles of HIF-PHIs.

There are still a few limitations to be mentioned. First, the analysis in this review heavily relied on the quality of reported results; however, none of the included meta-analyses were rated as high confidence on the basis of the methodological quality evaluation using the AMSTAR 2 system. Many included meta-analyses had not assessed the potential impact of risk of bias in individual primary studies on the evidence synthesis and thus were considered to have critical flaws. Future results from meta-analyses strictly adhering to the AMSTAR 2 system might help to procure better evidence for clinical decision making. Second, roxadustat and daprodustat were investigated more often than the other types of HIF-PHIs, such as enarodustat, vadadustat, and molidustat, inadvertently increasing the weights of roxadustat and daprodustat in the pooled results. The causes of this discrepancy may lie in the fact that roxadustat and daprodustat had longer research and development periods. More investigations on different types of HIF-PHIs would enable the full picture to be unveiled.

## Conclusion

To summarize, this is the first umbrella review examining the effectiveness and safety of HIF-PHIs in the treatment of renal anemia. The results demonstrate that HIF-PHI treatment successfully raises hemoglobin levels in CKD patients and enhances iron metabolism by reducing hepcidin levels and facilitating iron transport. The safety profiles of HIF-PHIs are generally comparable to those of ESA therapy or placebo.
