# Supplementary material for: Efficacy and safety of hypoxia-inducible factor-prolyl hydroxylase inhibitor treatment for anemia in chronic kidney disease: an umbrella review of meta-analyses
Source: Front Pharmacol. 2023 Nov 30;14:1296702. doi: 10.3389/fphar.2023.1296702 (PMC10720324; doi:10.3389/fphar.2023.1296702)
Supplement: Supplementary file 1 [file DataSheet1.docx]

**Supplementary Materials**

**Tables of contents**

[Supplementary Table 1. Literature search strategy used in this umbrella review. 2](#_Toc138022924)

[Supplementary Table 2: Justification for excluded publications after exclusion of duplicates during literature screening. 3](#_Toc138022925)

[Supplementary Table 3. Overall and individual scoring of the components of NutriGrade for each comparison of the efficacy and safety outcomes. 4](#_Toc138022926)

[Supplementary Table 4. Assessment of methodological quality with AMSTAR 2 tool for each comparison of the efficacy and safety outcomes. 9](#_Toc138022927)

[Supplementary Table 5. Efficacy of HIF-PHIs treatment on hemoglobin level compared with erythropoietin replacement or placebo. 14](#_Toc138022928)

[Supplementary Table 6. Efficacy of HIF-PHIs treatment on serum hepcidin level compared with erythropoietin replacement or placebo. 15](#_Toc138022929)

[Supplementary Table 7. Efficacy of HIF-PHIs treatment on serum transferrin compared with erythropoietin replacement or placebo. 16](#_Toc138022930)

[Supplementary Table 8. Efficacy of HIF-PHIs treatment on TIBC compared with erythropoietin replacement or placebo. 17](#_Toc138022931)

[Supplementary Table 9. Efficacy of HIF-PHIs treatment on TSAT compared with erythropoietin replacement or placebo. 18](#_Toc138022932)

[Supplementary Table 10. Efficacy of HIF-PHIs treatment on serum iron compared with erythropoietin replacement or placebo. 19](#_Toc138022933)

[Supplementary Table 11. Efficacy of HIF-PHIs treatment on serum ferritin compared with erythropoietin replacement or placebo. 20](#_Toc138022934)

[Supplementary Table 12. Efficacy of HIF-PHIs treatment on safety outcomes compared with erythropoietin replacement or placebo. 21](#_Toc138022935)

[Supplementary Figure 1. Efficacy of HIF-PHIs treatment on serum iron and ferritin compared with erythropoietin replacement or placebo. 23](#_Toc138022936)

[References 24](#_Toc138022937)

Supplementary Table 1. Literature search strategy used in this umbrella review.

| Database | Strategies |
| --- | --- |
| Embase via Ovid | 1. HIF-prolyl hydroxylase inhibitor*.mp.  2. Hypoxia-inducible factor.mp.  3. prolyl hydroxylase inhibitor*.mp.  4. HIF-PH inhibitor*.mp.  5. HIF-PHDi.mp.  6. kidney disease.mp.  7. nephropathy.mp.  8. renal disease.mp.  9. anemia/  10. 1 or 2 or 3 or 4 or 5  11. 6 or 7 or 8  12. 9 and 10 and 11  13. systematic review*.mp.  14. meta-analys*.mp.  15. 13 or 14  16. 12 and 15 |
| Medline via Pubmed | ((HIF-prolyl hydroxylase inhibitor*) OR (Hypoxia-inducible factor) OR (prolyl hydroxylase inhibitor*) OR (HIF-PH inhibitor*) OR (HIF-PHDi)) AND ((kidney disease) OR (nephropathy) OR (renal disease)) AND (anemia) AND ((systematic review*) OR (meta-analys*)) |
| Cochrane database of systematic reviews | ((HIF-prolyl hydroxylase inhibitor*) OR (Hypoxia-inducible factor) OR (prolyl hydroxylase inhibitor*) OR (HIF-PH inhibitor*) OR (HIF-PHDi)) AND ((kidney disease) OR (nephropathy) OR (renal disease)) AND (anemia) AND ((systematic review*) OR (meta-analys*)) |

Supplementary Table 2: Justification for excluded publications after exclusion of duplicates during literature screening.

| No. of studies excluded | Reasons for exclusion |
| --- | --- |
| 2 | Comments/Editorials^1,2^ |
| 3 | Protocols^3-5^ |
| 9 | Irrelevant interventions for renal anemia^6-14^ |
| 3 | Studies in non-CKD populations ^15-17^ |
| 5 | Abstract with insufficient information^18-22^ |
| 13 | Reviews without meta-analysis/not umbrella review ^23-35^ |
| 3 | Irrelevant outcomes^36-38^ |
| 5 | Network meta-analysis without extractable information for comparison^39-43^ |
| 6 | IPD meta-analyses^44-49^ |
| 3 | No summary estimates reported^50-52^ |
| 11 | Meta-analyses on the same topic^53-63^ |

Abbreviations: CKD, chronic kidney disease; IPD, individual patient data; No, number.

Supplementary Table 3. Overall and individual scoring of the components of NutriGrade for each comparison of the efficacy and safety outcomes.

| **Control** | **Experimental** | **Eligible MAs** | **Included MA** | **Risk of bias** | **Precision** | **Heterogeneity** | **Directness** | **Publication bias** | **Funding bias** | **Study Design** | **Sum** | **Quality** |
| --- | --- | --- | --- | --- | --- | --- | --- | --- | --- | --- | --- | --- |
| **Hemoglobulin** | | | | | | | | | | | | |
| EPO or Placebo | HIF-PHIs | 3 | Takkavatakarn 2023^64^ | 2.75 | 1 | 1 | 1 | 1 | 1 | 2 | 9.75 | High |
|  | Roxadustat | 4 | Liu 2020^65^ | 2.5 | 1 | 0.4 | 1 | 0.5 | 1 | 2 | 8.4 | High |
| EPO | HIF-PHIs | 4 | Mohamed 2023^66^ | 0 | 1 | 0.4 | 1 | 0 | 1 | 2 | 5.4 | Low |
|  | Roxadustat | 4 | Lei 2022^67^ | 3 | 1 | 0.5 | 1 | 0.5 | 1 | 2 | 9 | High |
|  | Daprodustat | 2 | Fatima 2022^68^ | 2.75 | 1 | 0.5 | 1 | 0 | 1 | 2 | 8.25 | High |
|  | Vadadustat | 1 | Xiong 2021^69^ | 3 | 1 | 0.5 | 1 | 1 | 1 | 2 | 9.5 | High |
|  | Molidustat | 1 | Wang 2020^70^ | 2.5 | 0 | 0.4 | 1 | 0 | 1 | 2 | 6.9 | Moderate |
| Placebo | HIF-PHIs | 5 | Wang 2020^70^ | 2.5 | 0 | 0.4 | 1 | 0 | 1 | 2 | 6.9 | Moderate |
|  | Roxadustat | 5 | Lei 2022^67^ | 3 | 1 | 0.5 | 1 | 0.5 | 1 | 2 | 9 | High |
|  | Daprodustat | 1 | Wang 2020^70^ | 2.5 | 0 | 0.4 | 1 | 0 | 1 | 2 | 6.9 | Moderate |
|  | Vadadustat | 1 | Xiong 2021^69^ | 3 | 0 | 0.5 | 1 | 1 | 1 | 2 | 8.5 | High |
|  | Molidustat | 1 | Wang 2020^70^ | 2.5 | 0 | 0.2 | 1 | 0 | 1 | 2 | 6.7 | Moderate |
|  | Desidustat | 1 | Wang 2020^70^ | 2.5 | 0 | 0.2 | 1 | 0 | 1 | 2 | 6.7 | Moderate |
| **Hepcidin** | | | | | | | | | | | | |
| EPO or Placebo | HIF-PHIs | 3 | Takkavatakarn 2023^64^ | 2.5 | 1 | 1 | 1 | 1 | 1 | 2 | 9.5 | High |
|  | Roxadustat | 5 | Zheng 2021^71^ | 2.25 | 1 | 0.5 | 1 | 0.5 | 1 | 2 | 8.25 | High |
| EPO | HIF-PHIs | 4 | Takkavatakarn 2023^64^ | 2.5 | 1 | 1 | 1 | 1 | 1 | 2 | 9.5 | High |
|  | Roxadustat | 7 | Zheng 2023^72^ | 1.75 | 1 | 0.5 | 1 | 0.5 | 1 | 2 | 7.75 | Moderate |
|  | Daprodustat | 4 | Fatima 2022^68^ | 2.25 | 1 | 0.5 | 1 | 0 | 1 | 2 | 7.75 | Moderate |
|  | Vadadustat | 2 | Zheng 2023^72^ | 1.75 | 1 | 0 | 1 | 0 | 1 | 2 | 6.75 | Moderate |
|  | Molidustat | 3 | Zheng 2023^72^ | 1.75 | 0 | 0 | 1 | 0 | 1 | 2 | 5.75 | Low |
|  | Enarodustat | 3 | Zheng 2023^72^ | 1.75 | 0 | 0 | 1 | 0 | 1 | 2 | 5.75 | Low |
|  | Desidustat | 2 | Zheng 2023^72^ | 1.75 | 0 | 0 | 1 | 0 | 1 | 2 | 5.75 | Low |
| Placebo | HIF-PHIs | 3 | Takkavatakarn 2023^64^ | 2.75 | 1 | 1 | 1 | 1 | 1 | 2 | 9.75 | High |
|  | Roxadustat | 5 | Zheng 2021^71^ | 2.25 | 1 | 0 | 1 | 0.5 | 1 | 2 | 7.75 | Moderate |
|  | Daprodustat | 3 | Li Jing 2021^73^ | 1.5 | 0 | 0 | 1 | 0 | 0 | 2 | 4.5 | Low |
|  | Vadadustat | 2 | Li Jing 2021^73^ | 2.5 | 0 | 0 | 1 | 0 | 0 | 2 | 5.5 | Low |
|  | Molidustat | 1 | Wang 2020^70^ | 2 | 0 | 0 | 1 | 0 | 1 | 2 | 6 | Moderate |
|  | Enarodustat | 1 | Wang 2020^70^ | 3 | 0 | 0 | 1 | 0 | 1 | 2 | 7 | Moderate |
|  | Desidustat | 1 | Wang 2020^70^ | 2.75 | 0 | 0 | 1 | 0 | 1 | 2 | 6.75 | Moderate |
| **Transferrin** | | | | | | | | | | | | |
| EPO or Placebo | HIF-PHIs | 1 | Wang 2020^70^ | 2.5 | 0 | 0.8 | 1 | 1 | 1 | 2 | 8.3 | High |
|  | Roxadustat | 4 | Zheng 2021^71^ | 2.5 | 1 | 0 | 1 | 0 | 1 | 2 | 7.5 | Moderate |
| EPO | HIF-PHIs | 2 | Zheng 2023^72^ | 1.75 | 1 | 0 | 1 | 0.5 | 1 | 2 | 7.25 | Moderate |
|  | Roxadustat | 6 | Zheng 2023^72^ | 1.75 | 1 | 0 | 1 | 0 | 1 | 2 | 6.75 | Moderate |
|  | Daprodustat | 1 | Zheng 2023^72^ | 1.75 | 0 | 0 | 1 | 0 | 1 | 2 | 5.75 | Low |
| placebo | HIF-PHIs | 1 | Wang 2020^70^ | 2.5 | 0 | 0.4 | 1 | 0.5 | 1 | 2 | 7.4 | Moderate |
|  | Roxadustat | 4 | Zheng 2021^71^ | 2.75 | 0 | 0 | 1 | 0 | 1 | 2 | 6.75 | Moderate |
| **TIBC** | | | | | | | | | | | | |
| EPO or Placebo | HIF-PHIs | 3 | Takkavatakarn 2023^64^ | 2.5 | 1 | 1 | 1 | 1 | 1 | 2 | 9.5 | High |
|  | Roxadustat | 7 | Zheng 2021^71^ | 2 | 1 | 0.5 | 1 | 0.5 | 1 | 2 | 8 | High |
| EPO | HIF-PHIs | 3 | Takkavatakarn 2023^64^ | 2.5 | 1 | 1 | 1 | 1 | 1 | 2 | 9.5 | High |
|  | Roxadustat | 7 | Zheng 2023^72^ | 1.75 | 1 | 0.5 | 1 | 0.5 | 1 | 2 | 7.75 | Moderate |
|  | Daprodustat | 3 | Fatima 2022^68^ | 2.75 | 1 | 0.5 | 1 | 0 | 1 | 2 | 8.25 | High |
|  | Vadadustat | 2 | Zheng 2023^72^ | 1.75 | 0 | 0 | 1 | 0 | 1 | 2 | 5.75 | Low |
|  | Molidustat | 2 | Zheng 2023^72^ | 1.75 | 0 | 0 | 1 | 0 | 1 | 2 | 5.75 | Low |
|  | Enarodustat | 2 | Zheng 2023^72^ | 1.75 | 0 | 0 | 1 | 0 | 1 | 2 | 5.75 | Low |
| Placebo | HIF-PHIs | 3 | Takkavatakarn 2023^64^ | 2.75 | 1 | 1 | 1 | 1 | 1 | 2 | 9.75 | High |
|  | Roxadustat | 5 | Zheng 2021^71^ | 2.25 | 1 | 0 | 1 | 0 | 1 | 2 | 7.25 | Moderate |
|  | Daprodustat | 1 | Li Jing 2021^73^ | 1.5 | 0 | 0 | 1 | 0 | 0 | 2 | 4.5 | Low |
|  | Vadadustat | 1 | Li Jing 2021^73^ | 2.5 | 0 | 0 | 1 | 0 | 0 | 2 | 5.5 | Low |
| **TSAT** | | | | | | | | | | | | |
| EPO or placebo | HIF-PHIs | 2 | Takkavatakarn 2023^64^ | 2.75 | 1 | 1 | 1 | 0 | 1 | 2 | 8.75 | High |
|  | Roxadustat | 7 | Zheng 2021^71^ | 2 | 1 | 0.5 | 1 | 0.5 | 1 | 2 | 8 | High |
| EPO | HIF-PHIs | 3 | Takkavatakarn 2023^64^ | 2.5 | 1 | 1 | 1 | 1 | 1 | 2 | 9.5 | High |
|  | Roxadustat | 7 | Zheng 2023^72^ | 1.75 | 1 | 0.5 | 1 | 0.5 | 1 | 2 | 7.75 | Moderate |
|  | Daprodustat | 3 | Fatima 2022^68^ | 2.75 | 1 | 0.5 | 1 | 0 | 1 | 2 | 8.25 | High |
|  | Vadadustat | 2 | Zheng 2023^72^ | 1.75 | 1 | 0 | 1 | 0 | 1 | 2 | 6.75 | Moderate |
|  | Molidustat | 2 | Zheng 2023^72^ | 1.75 | 1 | 0 | 1 | 0 | 1 | 2 | 6.75 | Moderate |
|  | Enarodustat | 2 | Zheng 2023^72^ | 1.75 | 0 | 0 | 1 | 0 | 1 | 2 | 5.75 | Low |
|  | Desidustat | 2 | Zheng 2023^72^ | 1.75 | 0 | 0 | 1 | 0 | 1 | 2 | 5.75 | Low |
| placebo | HIF-PHIs | 2 | Takkavatakarn 2023^64^ | 2.75 | 1 | 1 | 1 | 1 | 1 | 2 | 9.75 | High |
|  | Roxadustat | 2 | Zheng 2021^71^ | 2.25 | 1 | 0 | 1 | 0.5 | 1 | 2 | 7.75 | Moderate |
|  | Daprodustat | 2 | Zheng 2021^71^ | 1.75 | 0 | 0.4 | 1 | 0 | 1 | 2 | 6.15 | Moderate |
| **Serum Iron** | | | | | | | | | | | | |
| EPO or placebo | HIF-PHIs | 2 | Takkavatakarn 2023^64^ | 2.5 | 1 | 1 | 1 | 1 | 1 | 2 | 9.5 | High |
|  | Roxadustat | 5 | Zheng 2021^71^ | 2 | 0 | 0 | 1 | 0.5 | 1 | 2 | 6.5 | Moderate |
| EPO | HIF-PHIs | 3 | Takkavatakarn 2023^64^ | 2.5 | 1 | 1 | 1 | 1 | 1 | 2 | 9.5 | High |
|  | Roxadustat | 6 | Zheng 2023^72^ | 1.75 | 1 | 0.5 | 1 | 0.5 | 1 | 2 | 7.75 | Moderate |
|  | Daprodustat | 3 | Fatima 2022^68^ | 2.75 | 1 | 0.5 | 1 | 0 | 1 | 2 | 8.25 | High |
| EPO | Molidustat | 2 | Zheng 2023^72^ | 1.75 | 0 | 0 | 1 | 0 | 1 | 2 | 5.75 | Low |
|  | Enarodustat | 2 | Zheng 2023^72^ | 1.75 | 0 | 0 | 1 | 0 | 1 | 2 | 5.75 | Low |
|  | Desidustat | 2 | Zheng 2023^72^ | 1.75 | 0 | 0 | 1 | 0 | 1 | 2 | 5.75 | Low |
| placebo | HIF-PHIs | 3 | Takkavatakarn 2023^64^ | 2.75 | 1 | 1 | 1 | 1 | 1 | 2 | 9.75 | High |
|  | Roxadustat | 5 | Zheng 2021^71^ | 2 | 0 | 0 | 1 | 0 | 1 | 2 | 6 | Moderate |
|  | Daprodustat | 1 | Li Jing 2021^73^ | 2 | 0 | 0 | 1 | 0 | 0 | 2 | 5 | Low |
| **Serum Ferritin** | | | | | | | | | | | | |
| EPO or placebo | HIF-PHIs | 3 | Takkavatakarn 2023^64^ | 2.5 | 1 | 1 | 1 | 1 | 1 | 2 | 9.5 | High |
|  | Roxadustat | 5 | Zheng 2021^71^ | 2 | 1 | 0.5 | 1 | 0.5 | 1 | 2 | 8 | High |
| EPO | HIF-PHIs | 3 | Takkavatakarn 2023^64^ | 2.5 | 1 | 1 | 1 | 1 | 1 | 2 | 9.5 | High |
|  | Roxadustat | 7 | Zheng 2023^72^ | 1.75 | 1 | 0.5 | 1 | 0.5 | 1 | 2 | 7.75 | Moderate |
|  | Daprodustat | 3 | Fatima 2022^68^ | 2.75 | 1 | 0.5 | 1 | 0 | 1 | 2 | 8.25 | High |
|  | Vadadustat | 2 | Zheng 2023^72^ | 1.75 | 1 | 0 | 1 | 0 | 1 | 2 | 6.75 | Moderate |
|  | Molidustat | 2 | Zheng 2023^72^ | 1.75 | 0 | 0 | 1 | 0 | 1 | 2 | 5.75 | Low |
|  | Enarodustat | 2 | Zheng 2023^72^ | 1.75 | 0 | 0 | 1 | 0 | 1 | 2 | 5.75 | Low |
|  | Desidustat | 2 | Zheng 2023^72^ | 1.75 | 0 | 0 | 1 | 0 | 1 | 2 | 5.75 | Low |
| placebo | HIF-PHIs | 3 | Takkavatakarn 2023^64^ | 2.75 | 1 | 1 | 1 | 1 | 1 | 2 | 9.75 | High |
|  | Roxadustat | 5 | Zheng 2021^71^ | 2.25 | 1 | 0 | 1 | 0 | 1 | 2 | 7.25 | Moderate |
|  | Daprodustat | 2 | Li Jing 2021^73^ | 1.5 | 0 | 0 | 1 | 0 | 0 | 2 | 4.5 | Low |
|  | Vadadustat | 1 | Li Jing 2021^73^ | 2.5 | 0 | 0 | 1 | 0 | 0 | 2 | 5.5 | Low |
| **Mortality** | | | | | | | | | | | | |
| EPO or Placebo | HIF-PHIs | 1 | Takkavatakarn 2023^64^ | 2.5 | 1 | 0 | 1 | 1 | 1 | 2 | 8.5 | High |
| EPO | HIF-PHIs | 1 | Mohamed 2023^66^ | 0 | 1 | 0.4 | 1 | 0 | 1 | 2 | 5.4 | Low |
|  | Roxadustat | 1 | Qie 2021^74^ | 1 | 1 | 0.4 | 1 | 0 | 1 | 2 | 6.4 | Moderate |
| EPO | Daprodustat | 1 | Fatima 2022^68^ | 2.75 | 0 | 0.5 | 1 | 0 | 1 | 2 | 7.25 | Moderate |
| **AE** | | | | | | | | | | | | |
| EPO | HIF-PHIs | 4 | Mohamed 2023^66^ | 0 | 1 | 0.4 | 1 | 0 | 1 | 2 | 5.4 | Low |
|  | Roxadustat | 5 | Liu 2021^75^ | 2.75 | 1 | 0.3 | 1 | 0.5 | 1 | 2 | 8.55 | High |
| Placebo | HIF-PHIs | 3 | Zhang 2021^76^ | 2.25 | 1 | 0.5 | 1 | 0.5 | 1 | 2 | 8.25 | High |
|  | Roxadustat | 5 | Liu 2021^75^ | 2.75 | 1 | 0.3 | 1 | 0.5 | 1 | 2 | 8.55 | High |
| **SAE** | | | | | | | | | | | | |
| EPO | HIF-PHIs | 4 | Takkavatakarn 2023^64^ | 2.5 | 1 | 0 | 1 | 1 | 1 | 2 | 8.5 | High |
|  | Roxadustat | 2 | Zheng 2021^71^ | 2.5 | 1 | 0.5 | 1 | 0.5 | 1 | 2 | 8.5 | High |
| Placebo | HIF-PHIs | 1 | Wu 2022^77^ | 2 | 0.5 | 0.4 | 1 | 1 | 1 | 2 | 7.9 | Moderate |
|  | Roxadustat | 3 | Zheng 2021^71^ | 2.5 | 0.5 | 0.5 | 1 | 0.5 | 1 | 2 | 8 | High |
| **MACE** | | | | | | | | | | | | |
| EPO or Placebo | HIF-PHIs | 1 | Takkavatakarn 2023^64^ | 2.5 | 1 | 0 | 1 | 1 | 1 | 2 | 8.5 | High |
| EPO | HIF-PHIs | 4 | Mohamed 2023^66^ | 0 | 1 | 0.4 | 1 | 0 | 1 | 2 | 5.4 | Low |
|  | Roxadustat | 1 | Qie 2021^74^ | 1 | 1 | 0 | 1 | 0 | 1 | 2 | 6 | Moderate |
|  | Daprodustat | 1 | Fatima 2022^68^ | 2.75 | 1 | 0.5 | 1 | 0 | 1 | 2 | 8.25 | High |
| Placebo | HIF-PHIs | 1 | Wang 2020^70^ | 2.5 | 0 | 0.2 | 1 | 0 | 1 | 2 | 6.7 | Moderate |
|  | Roxadustat | 2 | Qie 2021^74^ | 1 | 0 | 0 | 1 | 0 | 1 | 2 | 5 | Low |
| **Stroke** | | | | | | | | | | | | |
| EPO or Placebo | HIF-PHIs | 1 | Takkavatakarn 2023^64^ | 2.5 | 1 | 0 | 1 | 1 | 1 | 2 | 8.5 | High |
| EPO | Roxadustat | 1 | Qie 2021^74^ | 1 | 0 | 0 | 1 | 0 | 1 | 2 | 5 | Low |
|  | Daprodustat | 1 | Fatima 2022^68^ | 2.75 | 0 | 0.5 | 1 | 0 | 1 | 2 | 7.25 | Moderate |

Abbreviations: AE, adverse events; EPO, erythropoietin; HIF-PHIs, hypoxia-inducible factor-prolyl hydroxylase inhibitors; MA, meta-analysis; MACE: major adverse cardiovascular events; SAE, severe adverse events TIBC, total iron binding capacity; TSAT, transferrin saturation.

Supplementary Table 4. Assessment of methodological quality with AMSTAR 2 tool for each comparison of the efficacy and safety outcomes.

| **Control** | **Experimental** | **Eligible MAs** | **Included MA** | **Q1** | **Q2** | **Q3** | **Q4** | **Q5** | **Q6** | **Q7** | **Q8** | **Q9** | **Q10** | **Q11** | **Q12** | **Q13** | **Q14** | **Q15** | **Q16** | **Overall Quality** |
| --- | --- | --- | --- | --- | --- | --- | --- | --- | --- | --- | --- | --- | --- | --- | --- | --- | --- | --- | --- | --- |
| **Hemoglobulin** | | | | | | | | | | | | | | | | | | | | |
| EPO or Placebo | HIF-PHIs | 3 | Takkavatakarn 2023^64^ | yes 1 | yes 1 | yes 1 | yes 1 | yes 1 | yes 1 | yes 1 | yes 1 | yes 1 | no 0 | yes 1 | no 0 | yes 1 | yes 1 | yes 1 | yes 1 | Moderate |
|  | Roxadustat | 4 | Liu 2020^65^ | yes 1 | yes 1 | yes 1 | yes 1 | yes 1 | yes 1 | yes 1 | yes 1 | yes 1 | no 0 | yes 1 | no 0 | yes 1 | yes 1 | yes 1 | yes 1 | Moderate |
| EPO | HIF-PHIs | 4 | Mohamed 2023^66^ | yes 1 | yes 1 | yes 1 | yes 1 | yes 1 | yes 1 | no 0 | 0.5 | no 0 | no 0 | yes 1 | no 0 | no 0 | no 0 | no 0 | yes 1 | Very low |
|  | Roxadustat | 4 | Lei 2022^67^ | yes 1 | yes 1 | yes 1 | yes 1 | yes 1 | yes 1 | yes 1 | yes 1 | yes 1 | no 0 | yes 1 | no 0 | yes 1 | yes 1 | yes 1 | yes 1 | Moderate |
|  | Daprodustat | 2 | Fatima 2022^68^ | yes 1 | yes 1 | yes 1 | yes 1 | yes 1 | yes 1 | yes 1 | yes 1 | yes 1 | no 0 | yes 1 | yes 1 | yes 1 | yes 1 | no 0 | yes 1 | Moderate |
|  | Vadadustat | 1 | Xiong 2021^69^ | yes 1 | yes 1 | yes 1 | yes 1 | yes 1 | yes 1 | yes 1 | yes 1 | yes 1 | no 0 | yes 1 | no 0 | yes 1 | yes 1 | yes 1 | yes 1 | Moderate |
|  | Molidustat | 1 | Wang 2020^70^ | yes 1 | yes 1 | yes 1 | yes 1 | yes 1 | yes 1 | yes 1 | yes 1 | yes 1 | no 0 | yes 1 | yes 1 | no 0 | yes 1 | yes 1 | yes 1 | Low |
| Placebo | HIF-PHIs | 5 | Wang 2020^70^ | yes 1 | yes 1 | yes 1 | yes 1 | yes 1 | yes 1 | yes 1 | yes 1 | yes 1 | no 0 | yes 1 | yes 1 | no 0 | yes 1 | yes 1 | yes 1 | Low |
|  | Roxadustat | 5 | Lei 2022^67^ | yes 1 | yes 1 | yes 1 | yes 1 | yes 1 | yes 1 | yes 1 | yes 1 | yes 1 | no 0 | yes 1 | no 0 | yes 1 | yes 1 | yes 1 | yes 1 | Moderate |
|  | Daprodustat | 1 | Wang 2020^70^ | yes 1 | yes 1 | yes 1 | yes 1 | yes 1 | yes 1 | yes 1 | yes 1 | yes 1 | no 0 | yes 1 | yes 1 | no 0 | yes 1 | yes 1 | yes 1 | Low |
|  | Vadadustat | 1 | Xiong 2021^69^ | yes 1 | yes 1 | yes 1 | yes 1 | yes 1 | yes 1 | yes 1 | yes 1 | yes 1 | no 0 | yes 1 | no 0 | yes 1 | yes 1 | yes 1 | yes 1 | Moderate |
|  | Molidustat | 1 | Wang 2020^70^ | yes 1 | yes 1 | yes 1 | yes 1 | yes 1 | yes 1 | yes 1 | yes 1 | yes 1 | no 0 | yes 1 | yes 1 | no 0 | yes 1 | yes 1 | yes 1 | Low |
|  | Desidustat | 1 | Wang 2020^70^ | yes 1 | yes 1 | yes 1 | yes 1 | yes 1 | yes 1 | yes 1 | yes 1 | yes 1 | no 0 | yes 1 | yes 1 | no 0 | yes 1 | yes 1 | yes 1 | Low |
| **Hepcidin** | | | | | | | | | | | | | | | | | | | | |
| EPO or Placebo | HIF-PHIs | 3 | Takkavatakarn 2023^64^ | yes 1 | yes 1 | yes 1 | yes 1 | yes 1 | yes 1 | yes 1 | yes 1 | yes 1 | no 0 | yes 1 | no 0 | yes 1 | yes 1 | yes 1 | yes 1 | Moderate |
|  | Roxadustat | 5 | Zheng 2021^71^ | yes 1 | yes 1 | yes 1 | yes 1 | yes 1 | yes 1 | yes 1 | yes 1 | yes 1 | no 0 | yes 1 | no 0 | yes 1 | yes 1 | yes 1 | yes 1 | Moderate |
| EPO | HIF-PHIs | 4 | Takkavatakarn 2023^64^ | yes 1 | yes 1 | yes 1 | yes 1 | yes 1 | yes 1 | yes 1 | yes 1 | yes 1 | no 0 | yes 1 | no 0 | yes 1 | yes 1 | yes 1 | yes 1 | Moderate |
|  | Roxadustat | 7 | Zheng 2023^72^ | yes 1 | yes 1 | yes 1 | yes 1 | yes 1 | yes 1 | yes 1 | yes 1 | yes 1 | no 0 | yes 1 | no 0 | no 0 | yes 1 | yes 1 | yes 1 | Low |
|  | Daprodustat | 4 | Fatima 2022^68^ | yes 1 | yes 1 | yes 1 | yes 1 | yes 1 | yes 1 | yes 1 | yes 1 | yes 1 | no 0 | yes 1 | yes 1 | yes 1 | yes 1 | no 0 | yes 1 | Moderate |
|  | Vadadustat | 2 | Zheng 2023^72^ | yes 1 | yes 1 | yes 1 | yes 1 | yes 1 | yes 1 | yes 1 | yes 1 | yes 1 | no 0 | yes 1 | no 0 | no 0 | yes 1 | yes 1 | yes 1 | Low |
|  | Molidustat | 3 | Zheng 2023^72^ | yes 1 | yes 1 | yes 1 | yes 1 | yes 1 | yes 1 | yes 1 | yes 1 | yes 1 | no 0 | yes 1 | no 0 | no 0 | yes 1 | yes 1 | yes 1 | Low |
|  | Enarodustat | 3 | Zheng 2023^72^ | yes 1 | yes 1 | yes 1 | yes 1 | yes 1 | yes 1 | yes 1 | yes 1 | yes 1 | no 0 | yes 1 | no 0 | no 0 | yes 1 | yes 1 | yes 1 | Low |
|  | Desidustat | 2 | Zheng 2023^72^ | yes 1 | yes 1 | yes 1 | yes 1 | yes 1 | yes 1 | yes 1 | yes 1 | yes 1 | no 0 | yes 1 | no 0 | no 0 | yes 1 | yes 1 | yes 1 | Low |
| Placebo | HIF-PHIs | 3 | Takkavatakarn 2023^64^ | yes 1 | yes 1 | yes 1 | yes 1 | yes 1 | yes 1 | yes 1 | yes 1 | yes 1 | no 0 | yes 1 | no 0 | yes 1 | yes 1 | yes 1 | yes 1 | Moderate |
|  | Roxadustat | 5 | Zheng 2021^71^ | yes 1 | yes 1 | yes 1 | yes 1 | yes 1 | yes 1 | yes 1 | yes 1 | yes 1 | no 0 | yes 1 | no 0 | yes 1 | yes 1 | yes 1 | yes 1 | Moderate |
|  | Daprodustat | 3 | Li Jing 2021^73^ | yes 1 | yes 1 | yes 1 | yes 1 | yes 1 | yes 1 | yes 1 | yes 1 | yes 1 | no 0 | yes 1 | no 0 | yes 1 | yes 1 | yes 1 | yes 1 | Moderate |
|  | Vadadustat | 2 | Li Jing 2021^73^ | yes 1 | yes 1 | yes 1 | yes 1 | yes 1 | yes 1 | yes 1 | yes 1 | yes 1 | no 0 | yes 1 | no 0 | yes 1 | yes 1 | yes 1 | yes 1 | Moderate |
|  | Molidustat | 1 | Wang 2020^70^ | yes 1 | yes 1 | yes 1 | yes 1 | yes 1 | yes 1 | yes 1 | yes 1 | yes 1 | no 0 | yes 1 | yes 1 | no 0 | yes 1 | yes 1 | yes 1 | Low |
|  | Enarodustat | 1 | Wang 2020^70^ | yes 1 | yes 1 | yes 1 | yes 1 | yes 1 | yes 1 | yes 1 | yes 1 | yes 1 | no 0 | yes 1 | yes 1 | no 0 | yes 1 | yes 1 | yes 1 | Low |
|  | Desidustat | 1 | Wang 2020^70^ | yes 1 | yes 1 | yes 1 | yes 1 | yes 1 | yes 1 | yes 1 | yes 1 | yes 1 | no 0 | yes 1 | yes 1 | no 0 | yes 1 | yes 1 | yes 1 | Low |
| **Transferrin** | | | | | | | | | | | | | | | | | | | | |
| EPO or Placebo | HIF-PHIs | 1 | Wang 2020^70^ | yes 1 | yes 1 | yes 1 | yes 1 | yes 1 | yes 1 | yes 1 | yes 1 | yes 1 | no 0 | yes 1 | yes 1 | no 0 | yes 1 | yes 1 | yes 1 | Low |
|  | Roxadustat | 4 | Zheng 2021^71^ | yes 1 | yes 1 | yes 1 | yes 1 | yes 1 | yes 1 | yes 1 | yes 1 | yes 1 | no 0 | yes 1 | no 0 | yes 1 | yes 1 | yes 1 | yes 1 | Moderate |
| EPO | HIF-PHIs | 2 | Zheng 2023^72^ | yes 1 | yes 1 | yes 1 | yes 1 | yes 1 | yes 1 | yes 1 | yes 1 | yes 1 | no 0 | yes 1 | no 0 | no 0 | yes 1 | yes 1 | yes 1 | Low |
|  | Roxadustat | 6 | Zheng 2023^72^ | yes 1 | yes 1 | yes 1 | yes 1 | yes 1 | yes 1 | yes 1 | yes 1 | yes 1 | no 0 | yes 1 | no 0 | no 0 | yes 1 | yes 1 | yes 1 | Low |
|  | Daprodustat | 1 | Zheng 2023^72^ | yes 1 | yes 1 | yes 1 | yes 1 | yes 1 | yes 1 | yes 1 | yes 1 | yes 1 | no 0 | yes 1 | no 0 | no 0 | yes 1 | yes 1 | yes 1 | Low |
| placebo | HIF-PHIs | 1 | Wang 2020^70^ | yes 1 | yes 1 | yes 1 | yes 1 | yes 1 | yes 1 | yes 1 | yes 1 | yes 1 | no 0 | yes 1 | yes 1 | no 0 | yes 1 | yes 1 | yes 1 | Low |
|  | Roxadustat | 4 | Zheng 2021^71^ | yes 1 | yes 1 | yes 1 | yes 1 | yes 1 | yes 1 | yes 1 | yes 1 | yes 1 | no 0 | yes 1 | no 0 | yes 1 | yes 1 | yes 1 | yes 1 | Moderate |
| **TIBC** | | | | | | | | | | | | | | | | | | | | |
| EPO or Placebo | HIF-PHIs | 3 | Takkavatakarn 2023^64^ | yes 1 | yes 1 | yes 1 | yes 1 | yes 1 | yes 1 | yes 1 | yes 1 | yes 1 | no 0 | yes 1 | no 0 | yes 1 | yes 1 | yes 1 | yes 1 | Moderate |
|  | Roxadustat | 7 | Zheng 2021^71^ | yes 1 | yes 1 | yes 1 | yes 1 | yes 1 | yes 1 | yes 1 | yes 1 | yes 1 | no 0 | yes 1 | no 0 | yes 1 | yes 1 | yes 1 | yes 1 | Moderate |
| EPO | HIF-PHIs | 3 | Takkavatakarn 2023^64^ | yes 1 | yes 1 | yes 1 | yes 1 | yes 1 | yes 1 | yes 1 | yes 1 | yes 1 | no 0 | yes 1 | no 0 | yes 1 | yes 1 | yes 1 | yes 1 | Moderate |
|  | Roxadustat | 7 | Zheng 2023^72^ | yes 1 | yes 1 | yes 1 | yes 1 | yes 1 | yes 1 | yes 1 | yes 1 | yes 1 | no 0 | yes 1 | no 0 | no 0 | yes 1 | yes 1 | yes 1 | Low |
|  | Daprodustat | 3 | Fatima 2022^68^ | yes 1 | yes 1 | yes 1 | yes 1 | yes 1 | yes 1 | yes 1 | yes 1 | yes 1 | no 0 | yes 1 | yes 1 | yes 1 | yes 1 | no 0 | yes 1 | Moderate |
|  | Vadadustat | 2 | Zheng 2023^72^ | yes 1 | yes 1 | yes 1 | yes 1 | yes 1 | yes 1 | yes 1 | yes 1 | yes 1 | no 0 | yes 1 | no 0 | no 0 | yes 1 | yes 1 | yes 1 | Low |
|  | Molidustat | 2 | Zheng 2023^72^ | yes 1 | yes 1 | yes 1 | yes 1 | yes 1 | yes 1 | yes 1 | yes 1 | yes 1 | no 0 | yes 1 | no 0 | no 0 | yes 1 | yes 1 | yes 1 | Low |
|  | Enarodustat | 2 | Zheng 2023^72^ | yes 1 | yes 1 | yes 1 | yes 1 | yes 1 | yes 1 | yes 1 | yes 1 | yes 1 | no 0 | yes 1 | no 0 | no 0 | yes 1 | yes 1 | yes 1 | Low |
| Placebo | HIF-PHIs | 3 | Takkavatakarn 2023^64^ | yes 1 | yes 1 | yes 1 | yes 1 | yes 1 | yes 1 | yes 1 | yes 1 | yes 1 | no 0 | yes 1 | no 0 | yes 1 | yes 1 | yes 1 | yes 1 | Moderate |
|  | Roxadustat | 5 | Zheng 2021^71^ | yes 1 | yes 1 | yes 1 | yes 1 | yes 1 | yes 1 | yes 1 | yes 1 | yes 1 | no 0 | yes 1 | no 0 | yes 1 | yes 1 | yes 1 | yes 1 | Moderate |
|  | Daprodustat | 1 | Li Jing 2021^73^ | yes 1 | yes 1 | yes 1 | yes 1 | yes 1 | yes 1 | yes 1 | yes 1 | yes 1 | no 0 | yes 1 | no 0 | yes 1 | yes 1 | yes 1 | yes 1 | Moderate |
|  | Vadadustat | 1 | Li Jing 2021^73^ | yes 1 | yes 1 | yes 1 | yes 1 | yes 1 | yes 1 | yes 1 | yes 1 | yes 1 | no 0 | yes 1 | no 0 | yes 1 | yes 1 | yes 1 | yes 1 | Moderate |
| **TSAT** | | | | | | | | | | | | | | | | | | | | |
| EPO or placebo | HIF-PHIs | 2 | Takkavatakarn 2023^64^ | yes 1 | yes 1 | yes 1 | yes 1 | yes 1 | yes 1 | yes 1 | yes 1 | yes 1 | no 0 | yes 1 | no 0 | yes 1 | yes 1 | yes 1 | yes 1 | Moderate |
|  | Roxadustat | 7 | Zheng 2021^71^ | yes 1 | yes 1 | yes 1 | yes 1 | yes 1 | yes 1 | yes 1 | yes 1 | yes 1 | no 0 | yes 1 | no 0 | yes 1 | yes 1 | yes 1 | yes 1 | Moderate |
| EPO | HIF-PHIs | 3 | Takkavatakarn 2023^64^ | yes 1 | yes 1 | yes 1 | yes 1 | yes 1 | yes 1 | yes 1 | yes 1 | yes 1 | no 0 | yes 1 | no 0 | yes 1 | yes 1 | yes 1 | yes 1 | Moderate |
|  | Roxadustat | 7 | Zheng 2023^72^ | yes 1 | yes 1 | yes 1 | yes 1 | yes 1 | yes 1 | yes 1 | yes 1 | yes 1 | no 0 | yes 1 | no 0 | no 0 | yes 1 | yes 1 | yes 1 | Low |
|  | Daprodustat | 3 | Fatima 2022^68^ | yes 1 | yes 1 | yes 1 | yes 1 | yes 1 | yes 1 | yes 1 | yes 1 | yes 1 | no 0 | yes 1 | yes 1 | yes 1 | yes 1 | no 0 | yes 1 | Moderate |
|  | Vadadustat | 2 | Zheng 2023^72^ | yes 1 | yes 1 | yes 1 | yes 1 | yes 1 | yes 1 | yes 1 | yes 1 | yes 1 | no 0 | yes 1 | no 0 | no 0 | yes 1 | yes 1 | yes 1 | Low |
|  | Molidustat | 2 | Zheng 2023^72^ | yes 1 | yes 1 | yes 1 | yes 1 | yes 1 | yes 1 | yes 1 | yes 1 | yes 1 | no 0 | yes 1 | no 0 | no 0 | yes 1 | yes 1 | yes 1 | Low |
|  | Enarodustat | 2 | Zheng 2023^72^ | yes 1 | yes 1 | yes 1 | yes 1 | yes 1 | yes 1 | yes 1 | yes 1 | yes 1 | no 0 | yes 1 | no 0 | no 0 | yes 1 | yes 1 | yes 1 | Low |
|  | Desidustat | 2 | Zheng 2023^72^ | yes 1 | yes 1 | yes 1 | yes 1 | yes 1 | yes 1 | yes 1 | yes 1 | yes 1 | no 0 | yes 1 | no 0 | no 0 | yes 1 | yes 1 | yes 1 | Low |
| placebo | HIF-PHIs | 2 | Takkavatakarn 2023^64^ | yes 1 | yes 1 | yes 1 | yes 1 | yes 1 | yes 1 | yes 1 | yes 1 | yes 1 | no 0 | yes 1 | no 0 | yes 1 | yes 1 | yes 1 | yes 1 | Moderate |
|  | Roxadustat | 2 | Zheng 2021^71^ | yes 1 | yes 1 | yes 1 | yes 1 | yes 1 | yes 1 | yes 1 | yes 1 | yes 1 | no 0 | yes 1 | no 0 | yes 1 | yes 1 | yes 1 | yes 1 | Moderate |
|  | Daprodustat | 2 | Zheng 2021^71^ | yes 1 | yes 1 | yes 1 | yes 1 | yes 1 | yes 1 | yes 1 | yes 1 | yes 1 | yes 1 | yes 1 | no 0 | no 0 | no 0 | no 0 | yes 1 | Low |
| **Serum Iron** | | | | | | | | | | | | | | | | | | | | |
| EPO or placebo | HIF-PHIs | 2 | Takkavatakarn 2023^64^ | yes 1 | yes 1 | yes 1 | yes 1 | yes 1 | yes 1 | yes 1 | yes 1 | yes 1 | no 0 | yes 1 | no 0 | yes 1 | yes 1 | yes 1 | yes 1 | Moderate |
|  | Roxadustat | 5 | Zheng 2021^71^ | yes 1 | yes 1 | yes 1 | yes 1 | yes 1 | yes 1 | yes 1 | yes 1 | yes 1 | no 0 | yes 1 | no 0 | yes 1 | yes 1 | yes 1 | yes 1 | Moderate |
| EPO | HIF-PHIs | 3 | Takkavatakarn 2023^64^ | yes 1 | yes 1 | yes 1 | yes 1 | yes 1 | yes 1 | yes 1 | yes 1 | yes 1 | no 0 | yes 1 | no 0 | yes 1 | yes 1 | yes 1 | yes 1 | Moderate |
|  | Roxadustat | 6 | Zheng 2023^72^ | yes 1 | yes 1 | yes 1 | yes 1 | yes 1 | yes 1 | yes 1 | yes 1 | yes 1 | no 0 | yes 1 | no 0 | no 0 | yes 1 | yes 1 | yes 1 | Low |
|  | Daprodustat | 3 | Fatima 2022^68^ | yes 1 | yes 1 | yes 1 | yes 1 | yes 1 | yes 1 | yes 1 | yes 1 | yes 1 | no 0 | yes 1 | yes 1 | yes 1 | yes 1 | no 0 | yes 1 | Moderate |
| EPO | Molidustat | 2 | Zheng 2023^72^ | yes 1 | yes 1 | yes 1 | yes 1 | yes 1 | yes 1 | yes 1 | yes 1 | yes 1 | no 0 | yes 1 | no 0 | no 0 | yes 1 | yes 1 | yes 1 | Low |
|  | Enarodustat | 2 | Zheng 2023^72^ | yes 1 | yes 1 | yes 1 | yes 1 | yes 1 | yes 1 | yes 1 | yes 1 | yes 1 | no 0 | yes 1 | no 0 | no 0 | yes 1 | yes 1 | yes 1 | Low |
|  | Desidustat | 2 | Zheng 2023^72^ | yes 1 | yes 1 | yes 1 | yes 1 | yes 1 | yes 1 | yes 1 | yes 1 | yes 1 | no 0 | yes 1 | no 0 | no 0 | yes 1 | yes 1 | yes 1 | Low |
| placebo | HIF-PHIs | 3 | Takkavatakarn 2023^64^ | yes 1 | yes 1 | yes 1 | yes 1 | yes 1 | yes 1 | yes 1 | yes 1 | yes 1 | no 0 | yes 1 | no 0 | yes 1 | yes 1 | yes 1 | yes 1 | Moderate |
|  | Roxadustat | 5 | Zheng 2021^71^ | yes 1 | yes 1 | yes 1 | yes 1 | yes 1 | yes 1 | yes 1 | yes 1 | yes 1 | no 0 | yes 1 | no 0 | yes 1 | yes 1 | yes 1 | yes 1 | Moderate |
|  | Daprodustat | 1 | Li Jing 2021^73^ | yes 1 | yes 1 | yes 1 | yes 1 | yes 1 | yes 1 | yes 1 | yes 1 | yes 1 | no 0 | yes 1 | no 0 | yes 1 | yes 1 | yes 1 | yes 1 | Moderate |
| **Serum Ferritin** | | | | | | | | | | | | | | | | | | | | |
| EPO or placebo | HIF-PHIs | 3 | Takkavatakarn 2023^64^ | yes 1 | yes 1 | yes 1 | yes 1 | yes 1 | yes 1 | yes 1 | yes 1 | yes 1 | no 0 | yes 1 | no 0 | yes 1 | yes 1 | yes 1 | yes 1 | Moderate |
|  | Roxadustat | 5 | Zheng 2021^71^ | yes 1 | yes 1 | yes 1 | yes 1 | yes 1 | yes 1 | yes 1 | yes 1 | yes 1 | no 0 | yes 1 | no 0 | yes 1 | yes 1 | yes 1 | yes 1 | Moderate |
| EPO | HIF-PHIs | 3 | Takkavatakarn 2023^64^ | yes 1 | yes 1 | yes 1 | yes 1 | yes 1 | yes 1 | yes 1 | yes 1 | yes 1 | no 0 | yes 1 | no 0 | yes 1 | yes 1 | yes 1 | yes 1 | Moderate |
|  | Roxadustat | 7 | Zheng 2023^72^ | yes 1 | yes 1 | yes 1 | yes 1 | yes 1 | yes 1 | yes 1 | yes 1 | yes 1 | no 0 | yes 1 | no 0 | no 0 | yes 1 | yes 1 | yes 1 | Low |
|  | Daprodustat | 3 | Fatima 2022^68^ | yes 1 | yes 1 | yes 1 | yes 1 | yes 1 | yes 1 | yes 1 | yes 1 | yes 1 | no 0 | yes 1 | yes 1 | yes 1 | yes 1 | no 0 | yes 1 | Moderate |
|  | Vadadustat | 2 | Zheng 2023^72^ | yes 1 | yes 1 | yes 1 | yes 1 | yes 1 | yes 1 | yes 1 | yes 1 | yes 1 | no 0 | yes 1 | no 0 | no 0 | yes 1 | yes 1 | yes 1 | Low |
|  | Molidustat | 2 | Zheng 2023^72^ | yes 1 | yes 1 | yes 1 | yes 1 | yes 1 | yes 1 | yes 1 | yes 1 | yes 1 | no 0 | yes 1 | no 0 | no 0 | yes 1 | yes 1 | yes 1 | Low |
|  | Enarodustat | 2 | Zheng 2023^72^ | yes 1 | yes 1 | yes 1 | yes 1 | yes 1 | yes 1 | yes 1 | yes 1 | yes 1 | no 0 | yes 1 | no 0 | no 0 | yes 1 | yes 1 | yes 1 | Low |
|  | Desidustat | 2 | Zheng 2023^72^ | yes 1 | yes 1 | yes 1 | yes 1 | yes 1 | yes 1 | yes 1 | yes 1 | yes 1 | no 0 | yes 1 | no 0 | no 0 | yes 1 | yes 1 | yes 1 | Low |
| placebo | HIF-PHIs | 3 | Takkavatakarn 2023^64^ | yes 1 | yes 1 | yes 1 | yes 1 | yes 1 | yes 1 | yes 1 | yes 1 | yes 1 | no 0 | yes 1 | no 0 | yes 1 | yes 1 | yes 1 | yes 1 | Moderate |
|  | Roxadustat | 5 | Zheng 2021^71^ | yes 1 | yes 1 | yes 1 | yes 1 | yes 1 | yes 1 | yes 1 | yes 1 | yes 1 | no 0 | yes 1 | no 0 | yes 1 | yes 1 | yes 1 | yes 1 | Moderate |
|  | Daprodustat | 2 | Li Jing 2021^73^ | yes 1 | yes 1 | yes 1 | yes 1 | yes 1 | yes 1 | yes 1 | yes 1 | yes 1 | no 0 | yes 1 | no 0 | yes 1 | yes 1 | yes 1 | yes 1 | Moderate |
|  | Vadadustat | 1 | Li Jing 2021^73^ | yes 1 | yes 1 | yes 1 | yes 1 | yes 1 | yes 1 | yes 1 | yes 1 | yes 1 | no 0 | yes 1 | no 0 | yes 1 | yes 1 | yes 1 | yes 1 | Moderate |
| **Mortality** | | | | | | | | | | | | | | | | | | | | |
| EPO or Placebo | HIF-PHIs | 1 | Takkavatakarn 2023^64^ | yes 1 | yes 1 | yes 1 | yes 1 | yes 1 | yes 1 | yes 1 | yes 1 | yes 1 | no 0 | yes 1 | no 0 | yes 1 | yes 1 | yes 1 | yes 1 | Moderate |
| EPO | HIF-PHIs | 1 | Mohamed 2023^66^ | yes 1 | yes 1 | yes 1 | yes 1 | yes 1 | yes 1 | no 0 | 0.5 | no 0 | no 0 | yes 1 | no 0 | no 0 | no 0 | no 0 | yes 1 | Very low |
|  | Roxadustat | 1 | Qie 2021^74^ | yes 1 | yes 1 | yes 1 | yes 1 | yes 1 | yes 1 | yes 1 | yes 1 | yes 1 | no 0 | yes 1 | yes 1 | yes 1 | yes 1 | no 0 | yes 1 | Low |
| EPO | Daprodustat | 1 | Fatima 2022^68^ | yes 1 | yes 1 | yes 1 | yes 1 | yes 1 | yes 1 | yes 1 | yes 1 | yes 1 | no 0 | yes 1 | yes 1 | yes 1 | yes 1 | no 0 | yes 1 | Moderate |
| **AE** | | | | | | | | | | | | | | | | | | | | |
| EPO | HIF-PHIs | 4 | Mohamed 2023^66^ | yes 1 | yes 1 | yes 1 | yes 1 | yes 1 | yes 1 | no 0 | 0.5 | no 0 | no 0 | yes 1 | no 0 | no 0 | no 0 | no 0 | yes 1 | Very low |
|  | Roxadustat | 5 | Liu 2021^75^ | yes 1 | yes 1 | yes 1 | yes 1 | yes 1 | yes 1 | yes 1 | yes 1 | yes 1 | no 0 | yes 1 | no 0 | yes 1 | yes 1 | yes 1 | yes 1 | Moderate |
| Placebo | HIF-PHIs | 3 | Zhang 2021^76^ | yes 1 | yes 1 | yes 1 | yes 1 | yes 1 | yes 1 | yes 1 | yes 1 | yes 1 | yes 1 | yes 1 | no 0 | no 0 | no 0 | no 0 | yes 1 | Low |
|  | Roxadustat | 5 | Liu 2021^75^ | yes 1 | yes 1 | yes 1 | yes 1 | yes 1 | yes 1 | yes 1 | yes 1 | yes 1 | no 0 | yes 1 | no 0 | yes 1 | yes 1 | yes 1 | yes 1 | Moderate |
| **SAE** | | | | | | | | | | | | | | | | | | | | |
| EPO | HIF-PHIs | 4 | Takkavatakarn 2023^64^ | yes 1 | yes 1 | yes 1 | yes 1 | yes 1 | yes 1 | yes 1 | yes 1 | yes 1 | no 0 | yes 1 | no 0 | yes 1 | yes 1 | yes 1 | yes 1 | Moderate |
|  | Roxadustat | 2 | Zheng 2021^71^ | yes 1 | yes 1 | yes 1 | yes 1 | yes 1 | yes 1 | yes 1 | yes 1 | yes 1 | no 0 | yes 1 | no 0 | yes 1 | yes 1 | yes 1 | yes 1 | Moderate |
| Placebo | HIF-PHIs | 1 | Wu 2022^77^ | yes 1 | yes 1 | yes 1 | yes 1 | yes 1 | yes 1 | yes 1 | yes 1 | yes 1 | no 0 | yes 1 | no 0 | yes 1 | yes 1 | yes 1 | yes 1 | Moderate |
|  | Roxadustat | 3 | Zheng 2021^71^ | yes 1 | yes 1 | yes 1 | yes 1 | yes 1 | yes 1 | yes 1 | yes 1 | yes 1 | no 0 | yes 1 | no 0 | yes 1 | yes 1 | yes 1 | yes 1 | Moderate |
| **MACE** | | | | | | | | | | | | | | | | | | | | |
| EPO or Placebo | HIF-PHIs | 1 | Takkavatakarn 2023^64^ | yes 1 | yes 1 | yes 1 | yes 1 | yes 1 | yes 1 | yes 1 | yes 1 | yes 1 | no 0 | yes 1 | no 0 | yes 1 | yes 1 | yes 1 | yes 1 | Moderate |
| EPO | HIF-PHIs | 4 | Mohamed 2023^66^ | yes 1 | yes 1 | yes 1 | yes 1 | yes 1 | yes 1 | no 0 | 0.5 | no 0 | no 0 | yes 1 | no 0 | no 0 | no 0 | no 0 | yes 1 | Very low |
|  | Roxadustat | 1 | Qie 2021^74^ | yes 1 | yes 1 | yes 1 | yes 1 | yes 1 | yes 1 | yes 1 | yes 1 | yes 1 | no 0 | yes 1 | yes 1 | yes 1 | yes 1 | no 0 | yes 1 | Low |
|  | Daprodustat | 1 | Fatima 2022^68^ | yes 1 | yes 1 | yes 1 | yes 1 | yes 1 | yes 1 | yes 1 | yes 1 | yes 1 | no 0 | yes 1 | yes 1 | yes 1 | yes 1 | no 0 | yes 1 | Moderate |
| Placebo | HIF-PHIs | 1 | Wang 2020^70^ | yes 1 | yes 1 | yes 1 | yes 1 | yes 1 | yes 1 | yes 1 | yes 1 | yes 1 | no 0 | yes 1 | yes 1 | no 0 | yes 1 | yes 1 | yes 1 | Low |
|  | Roxadustat | 2 | Qie 2021^74^ | yes 1 | yes 1 | yes 1 | yes 1 | yes 1 | yes 1 | yes 1 | yes 1 | yes 1 | no 0 | yes 1 | yes 1 | yes 1 | yes 1 | no 0 | yes 1 | Low |
| **Stroke** | | | | | | | | | | | | | | | | | | | | |
| EPO or Placebo | HIF-PHIs | 1 | Takkavatakarn 2023^64^ | yes 1 | yes 1 | yes 1 | yes 1 | yes 1 | yes 1 | yes 1 | yes 1 | yes 1 | no 0 | yes 1 | no 0 | yes 1 | yes 1 | yes 1 | yes 1 | Moderate |
| EPO | Roxadustat | 1 | Qie 2021^74^ | yes 1 | yes 1 | yes 1 | yes 1 | yes 1 | yes 1 | yes 1 | yes 1 | yes 1 | no 0 | yes 1 | yes 1 | yes 1 | yes 1 | no 0 | yes 1 | Low |
|  | Daprodustat | 1 | Fatima 2022^68^ | yes 1 | yes 1 | yes 1 | yes 1 | yes 1 | yes 1 | yes 1 | yes 1 | yes 1 | no 0 | yes 1 | yes 1 | yes 1 | yes 1 | no 0 | yes 1 | Moderate |

Abbreviations: AE, adverse events; EPO, erythropoietin; HIF-PHIs, hypoxia-inducible factor-prolyl hydroxylase inhibitors; MA, meta-analysis; MACE: major adverse cardiovascular events; SAE, severe adverse events TIBC, total iron binding capacity; TSAT, transferrin saturation.

Supplementary Table 5. Efficacy of HIF-PHIs treatment on hemoglobin level compared with erythropoietin replacement or placebo.

| **Experimental** | **Total eligible MAs** | **Included MA** | **Targeted population** | **No. of primary studies** | **No. of patients** | **MA metrics** | **Estimates (95% CI)** | **Effects model** | **P-value** | **I^2^ (%)** | **P-value of Q test** | **NutriGrade** | **AMSTAR2** |
| --- | --- | --- | --- | --- | --- | --- | --- | --- | --- | --- | --- | --- | --- |
| **HIF-PHIs versus EPO or Placebo** | | | | | | | | | | | | | |
| HIF-PHIs | 3 | Takkavatakarn 2023^64^ | DD+NDD | 46 | 27338 | MD | 0.66 (0.50, 0.82) | Random | <0.001 | 98.9 | <0.001 | High | Moderate |
| Roxadustat | 4 | Liu 2020^65^ | DD+NDD | 6 | 1295 | MD | 1.20 (0.66, 1.75) | Random | <0.001 | 99.3 | <0.001 | High | Moderate |
| **HIF-PHIs versus EPO** | | | | | | | | | | | | | |
| HIF-PHIs | 4 | Mohamed 2023^66^ | NDD | 7 | 8228 | MD | -0.03 (-0.02, 0.11) | Random | 0.66 | 90 | <0.001 | Low | Very low |
| Roxadustat | 4 | Lei 2022^67^ | DD | 9 | 3721 | SMD | 0.21 (-0.10, 0.52) | Random | 0.18 | 95 | <0.001 | High | Moderate |
| Daprodustat | 2 | Fatima 2022^68^ | DD+NDD | 8 | 8157 | MD | 0.05 (-0.10, 0.21) | Random | 0.5 | 96 | <0.001 | High | Moderate |
| Vadadustat | 1 | Xiong 2021^69^ | DD+NDD | 3 | 4171 | RR | 0.96 (0.90, 1.03) | Random | 0.29 | 68 | NR | High | Moderate |
| Molidustat | 1 | Wang 2020^70^ | DD+NDD | 4 | 640 | MD | 0.07 (-0.13, 0.26) | Random | NR | 0 | 0.6 | Moderate | Low |
| HIF-PHIs versus Placebo | | | | | | | | | | | | | |
| HIF-PHIs | 5 | Wang 2020^70^ | DD+NDD | 13 | 1897 | MD | 1.17 (0.79, 1.54) | Random | <0.001 | 94.3 | <0.001 | Moderate | Low |
| Roxadustat | 5 | Lei 2022^67^ | NDD | 5 | 1721 | SMD | 1.77 (1.52, 2.02) | Random | <0.001 | 69 | 0.01 | High | Moderate |
| Daprodustat | 1 | Wang 2020^70^ | DD+NDD | 4 | 748 | MD | 0.56 (-0.26, 1.37) | Random | NR | 94.2 | <0.001 | Moderate | Low |
| Vadadustat | 1 | Xiong 2021^69^ | NDD | 2 | 249 | RR | 5.27 (2.69, 10.31) | Random | <0.001 | 0 | NR | High | Moderate |
| Molidustat | 1 | Wang 2020^70^ | NDD | 1 | 120 | MD | 1.60 (0.88, 2.32) | Random | NR | NR | 1 | Moderate | Low |
| Desidustat | 1 | Wang 2020^70^ | NDD | 1 | 147 | MD | 1.78 (1.08, 2.47) | Random | NR | NR | <0.001 | Moderate | Low |

Abbreviations: EPO, erythropoietin; DD, dialysis dependent; HIF-PHIs, hypoxia-inducible factor-prolyl hydroxylase inhibitors; MA, meta-analysis; MD, mean difference; NDD, non-dialysis dependent; NR, not reported; RR: risk ratio; SMD, standardized mean difference.

Supplementary Table 6. Efficacy of HIF-PHIs treatment on serum hepcidin level compared with erythropoietin replacement or placebo.

| **Experimental** | **Total eligible MAs** | **Included MA** | **Targeted population** | **No. of primary studies** | **No. of patients** | **MA metrics** | **Estimates (95% CI)** | **Effects model** | **P-value** | **I^2^ (%)** | **P-value of Q test** | **NutriGrade** | **AMSTAR2** |
| --- | --- | --- | --- | --- | --- | --- | --- | --- | --- | --- | --- | --- | --- |
| **HIF-PHIs versus EPO or Placebo** | | | | | | | | | | | | | |
| HIF-PHIs | 3 | Takkavatakarn 2023^64^ | DD+NDD | 34 | 20270 | MD | -29.11 (-34.41, -23.81) | Random | <0.001 | 99.4 | <0.001 | High | Moderate |
| Roxadustat | 5 | Zheng 2021^71^ | DD+NDD | 8 | 2069 | MD | -23.16 (-37.12, -9.19) | Random | <0.00001 | 93 | 0.001 | High | Moderate |
| **HIF-PHIs versus EPO** | | | | | | | | | | | | | |
| HIF-PHIs | 4 | Takkavatakarn 2023^64^ | DD+NDD | 30 | 16046 | MD | -19.12 (-25.50, -12.74) | Random | <0.001 | 99.0 | <0.001 | High | Moderate |
| Roxadustat | 7 | Zheng 2023^72^ | DD | 9 | 4396 | MD | -17.95 (-28.96, -6.94) | Random | <0.001 | 79 | 0.001 | Moderate | Low |
| Daprodustat | 4 | Fatima 2022^68^ | DD+NDD | 8 | 8011 | MD | -26.30 (-34.20, -18.30) | Random | <0.0001 | 11 | 0.35 | Moderate | Moderate |
| Vadadustat | 2 | Zheng 2023^72^ | DD | 2 | 4246 | MD | -13.60 (-25.68, -1.52) | Random | 0.0008 | 86 | 0.03 | Moderate | Low |
| Molidustat | 3 | Zheng 2023^72^ | DD | 2 | 428 | MD | 2.70 (-3.37, 8.78) | Fixed | 0.79 | 0 | 0.38 | Low | Low |
| Enarodustat | 3 | Zheng 2023^72^ | DD | 1 | 172 | MD | -5.91 (-43.40, 31.60) | - | - | - | 0.76 | Low | Low |
| Desidustat | 2 | Zheng 2023^72^ | DD | 1 | 392 | MD | -17.40 (-42.00, 7.20) | - | - | - | 0.16 | Low | Low |
| **HIF-PHIs versus Placebo** | | | | | | | | | | | | | |
| HIF-PHIs | 3 | Takkavatakarn 2023^64^ | DD+NDD | 38 | 4224 | MD | -39.77 (-49.42, -30.12) | Random | <0.001 | 99.5 | <0.001 | High | Moderate |
| Roxadustat | 5 | Zheng 2021^71^ | NDD | 5 | 705 | MD | -29.50 (-44.46, -14.54) | Random | 0.0001 | 87 | <0.0001 | Moderate | Moderate |
| Daprodustat | 3 | Li Jing 2021^73^ | NDD | 2 | 56 | SMD | -0.76 (-1.38, -0.14) | Random | 0.02 | 0 | 0.39 | Low | Moderate |
| Vadadustat | 2 | Li Jing 2021^73^ | NDD | 2 | 297 | SMD | -0.54 (-0.79, -0.28) | Random | <0.0001 | 0 | 0.75 | Low | Moderate |
| Molidustat | 1 | Wang 2020^70^ | DD+NDD | 1 | NR | MD | -20.00 (-35.75, -4.25) | - | - | - |  | Moderate | Low |
| Enarodustat | 1 | Wang 2020^70^ | DD+NDD | 2 | NR | MD | -44.71 (-84.14, -5.29) | Random | 0.034 | 77.57 | - | Moderate | Low |
| Desidustat | 1 | Wang 2020^70^ | DD+NDD | 1 | NR | MD | -36.57 (-70.05, -3.09) | - | - | - |  | Moderate | Low |

Abbreviations: EPO, erythropoietin; DD, dialysis dependent; HIF-PHIs, hypoxia-inducible factor-prolyl hydroxylase inhibitors; MA, meta-analysis; MD, mean difference; NDD, non-dialysis dependent; NR, not reported; SMD, standardized mean difference.

Supplementary Table 7. Efficacy of HIF-PHIs treatment on serum transferrin compared with erythropoietin replacement or placebo.

| **Experimental** | **Total eligible MA** | **Included MA** | **Targeted population** | **No. of primary studies** | **No. of patients** | **MA metrics** | **Estimates (95% CI)** | **Effects model** | **P-value** | **I^2^ (%)** | **P-value of Q test** | **NutriGrade** | **AMSTAR2** |
| --- | --- | --- | --- | --- | --- | --- | --- | --- | --- | --- | --- | --- | --- |
| **HIF-PHIs versus EPO or Placebo** | | | | | | | | | | | | | |
| HIF-PHIs | 1 | Wang 2020^70^ | DD+NDD | 11 | NR | MD | 0.91 (0.52, 1.30) | Random | NR | 87.0 | <0.001 | High | Low |
| Roxadustat | 4 | Zheng 2021^71^ | DD+NDD | 4 | 987 | MD | 0.50 (0.34, 0.65) | Random | <0.00001 | 91 | <0.00001 | Moderate | Moderate |
| **HIF-PHIs versus EPO** | | | | | | | | | | | | | |
| HIF-PHIs | 2 | Zheng 2023^72^ | DD | 5 | 908 | SMD | 0.90 (0.74, 1.05) | Random | <0.001 | 15 | 0.32 | Moderate | Low |
| Roxadustat | 6 | Zheng 2023^72^ | DD | 3 | 637 | SMD | 0.98 (0.81, 1.15) | Fixed | <0.001 | 0 | 0.42 | Moderate | Low |
| Daprodustat | 1 | Zheng 2023^72^ | DD | 2 | 271 | SMD | 0.72 (0.47, 0.96) | Fixed | <0.001 | 15 | 0.32 | Low | Low |
| **HIF-PHIs versus Placebo** | | | | | | | | | | | | | |
| HIF-PHIs | 1 | Wang 2020^70^ | DD+NDD | 6 | NR | MD | 1.03 (0.34, 1.73) | Random | NR | 91.7 | <0.001 | Moderate | Low |
| Roxadustat | 4 | Zheng 2021^71^ | NDD | 3 | 350 | MD | 0.60 (0.25, 0.95) | Random | 0.0008 | 95 | <0.00001 | Moderate | Moderate |

Abbreviations: EPO, erythropoietin; DD, dialysis dependent; HIF-PHIs, hypoxia-inducible factor-prolyl hydroxylase inhibitors; MA, meta-analysis; MD, mean difference; NDD, non-dialysis dependent; NR, not reported; SMD, standardized mean difference.

Supplementary Table 8. Efficacy of HIF-PHIs treatment on TIBC compared with erythropoietin replacement or placebo.

| **Experimental** | **Total eligible MA** | **Included MA** | **Targeted population** | **No. of primary studies** | **No. of patients** | **MA metrics** | **Estimates (95% CI)** | **Effects model** | **P-value** | **I^2^ (%)** | **P-value of Q test** | **NutriGrade** | **AMSTAR2** |
| --- | --- | --- | --- | --- | --- | --- | --- | --- | --- | --- | --- | --- | --- |
| **HIF-PHIs versus EPO or Placebo** | | | | | | | | | | | | | |
| HIF-PHIs | 3 | Takkavatakarn 2023^64^ | DD+NDD | 30 | 9206 | MD | 43.81 (39.16, 48.46) | Random | <0.001 | 96.3 | <0.001 | High | Moderate |
| Roxadustat | 7 | Zheng 2021^71^ | DD+NDD | 6 | 1195 | MD | 50.64 (36.21, 65.07) | Random | <0.00001 | 88 | <0.00001 | High | Moderate |
| **HIF-PHIs versus EPO** | | | | | | | | | | | | | |
| HIF-PHIs | 3 | Takkavatakarn 2023^64^ | DD+NDD | 23 | 4063 | MD | 27.78 (19.79, 35.77) | Random | <0.001 | 93.7 | <0.001 | High | Moderate |
| Roxadustat | 7 | Zheng 2023^72^ | DD | 8 | 3701 | SMD | 0.88 (0.65, 1.11) | Random | <0.001 | 88 | <0.001 | Moderate | Low |
| Daprodustat | 3 | Fatima 2022^68^ | DD+NDD | 8 | 7870 | MD | 6.10 (4.60, 7.59) | Random | <0.0001 | 95 | <0.0001 | High | Moderate |
| Vadadustat | 2 | Zheng 2023^72^ | DD | 1 | 323 | SMD | 1.30 (1.06, 1.54) | - | - | - | <0.0001 | Low | Low |
| Molidustat | 2 | Zheng 2023^72^ | DD | 2 | 428 | SMD | 0.15 (-0.06, 0.37) | Fixed | 0.55 | 0 | 0.16 | Low | Low |
| Enarodustat | 2 | Zheng 2023^72^ | DD | 1 | 172 | SMD | 0.40 (0.10, 0.70) | - | - | - | 0.009 | Low | Low |
| **HIF-PHIs versus Placebo** | | | | | | | | | | | | | |
| HIF-PHIs | 3 | Takkavatakarn 2023^64^ | DD+NDD | 35 | 5143 | MD | 54.80 (48.35, 61.25) | Random | <0.001 | 96.8 | <0.001 | High | Moderate |
| Roxadustat | 5 | Zheng 2021^71^ | NDD | 4 | 435 | MD | 65.66 (35.47, 95.84) | Random | <0.0001 | 93 | <0.00001 | Moderate | Moderate |
| Daprodustat | 1 | Li Jing 2021^73^ | NDD | 2 | 262 | MD | 4.65 (1.86, 7.44) | Random | 0.05 | 61 | 0.001 | Low | Moderate |
| Vadadustat | 1 | Li Jing 2021^73^ | NDD | 2 | 297 | MD | 6.88 (4.43, 9.34) | Random | <0.00001 | 45 | 0.12 | Low | Moderate |

Abbreviations: EPO, erythropoietin; DD, dialysis dependent; HIF-PHIs, hypoxia-inducible factor-prolyl hydroxylase inhibitors; MA, meta-analysis; MD, mean difference; NDD, non-dialysis dependent; SMD, standardized mean difference; TIBC, total iron binding capacity.

Supplementary Table 9. Efficacy of HIF-PHIs treatment on TSAT compared with erythropoietin replacement or placebo.

| **Experimental** | **Total eligible MA** | **Included MA** | **Targeted population** | **No. of primary studies** | **No. of patients** | **MA metrics** | **Estimates (95% CI)** | **Effects model** | **P-value** | **I^2^ (%)** | **P-value of Q test** | **NutriGrade** | **AMSTAR2** |
| --- | --- | --- | --- | --- | --- | --- | --- | --- | --- | --- | --- | --- | --- |
| **HIF-PHIs versus EPO or Placebo** | | | | | | | | | | | | | |
| HIF-PHIs | 2 | Takkavatakarn 2023^64^ | DD+NDD | 38 | 22585 | MD | -3.00 (-3.67, -2.33) | Random | <0.0001 | 98.0 | <0.0001 | High | Moderate |
| Roxadustat | 7 | Zheng 2021^71^ | DD+NDD | 8 | 2218 | MD | -1.15 (-3.07, 0.78) | Random | 0.24 | 62 | 0.004 | High | Moderate |
| HIF-PHIs versus EPO | | | | | | | | | | | | | |
| HIF-PHIs | 3 | Takkavatakarn 2023^64^ | DD+NDD | 33 | 17048 | MD | -1.83 (-2.91, -0.74) | Random | 0.001 | 98.1 | <0.001 | High | Moderate |
| Roxadustat | 7 | Zheng 2023^72^ | DD | 9 | 4758 | MD | 0.82 (-0.51, 2.15) | Random | 0.23 | 68 | 0.001 | Moderate | Low |
| Daprodustat | 3 | Fatima 2022^68^ | DD+NDD | 8 | 7839 | MD | -5.35 (-12.06, 1.36) | Random | 0.12 | 98 | <0.0001 | High | Moderate |
| Vadadustat | 2 | Zheng 2023^72^ | DD | 2 | 4243 | MD | -0.48 (-3.55, 2.59) | Random | 0.76 | 95 | <0.001 | Moderate | Low |
| Molidustat | 2 | Zheng 2023^72^ | DD | 2 | 428 | MD | 3.97 (1.70, 6.24) | Fixed | 0.28 | 15 | 0.0006 | Moderate | Low |
| Enarodustat | 2 | Zheng 2023^72^ | DD | 1 | 172 | MD | 1.95 (-9.41, 13.31) | - | 0.74 | - | - | Low | Low |
| Desidustat | 2 | Zheng 2023^72^ | DD | 1 | 392 | MD | -2.40 (-5.62, 0.82) | - | 0.14 | - | - | Low | Low |
| HIF-PHIs versus Placebo | | | | | | | | | | | | | |
| HIF-PHIs | 2 | Takkavatakarn 2023^64^ | DD+NDD | 31 | 5537 | MD | -4.78 (-5.94, -3.62) | Random | <0.001 | 93.1 | <0.001 | High | Moderate |
| Roxadustat | 2 | Zheng 2021^71^ | NDD | 5 | 854 | MD | -3.81 (-5.43, -2.19) | Random | <0.0001 | 0 | 0.41 | Moderate | Moderate |
| Daprodustat | 2 | Zheng 2021^71^ | NDD | 6 | 568 | SMD | -0.23 (-0.66, 0.21) | Random | 0.31 | 80 | 0.0002 | Moderate | Low |

Abbreviations: EPO, erythropoietin; DD, dialysis dependent; HIF-PHIs, hypoxia-inducible factor-prolyl hydroxylase inhibitors; MA, meta-analysis; MD, mean difference; NDD, non-dialysis dependent; SMD, standardized mean difference; TSAT, transferrin saturation.

Supplementary Table 10. Efficacy of HIF-PHIs treatment on serum iron compared with erythropoietin replacement or placebo.

| **Experimental** | **Total eligible MA** | **Included MA** | **Targeted population** | **No. of primary studies** | **No. of patients** | **MA metrics** | **Estimates (95% CI)** | **Effects model** | **P-value** | **I^2^ (%)** | **P-value of Q test** | **NutriGrade** | **AMSTAR2** |
| --- | --- | --- | --- | --- | --- | --- | --- | --- | --- | --- | --- | --- | --- |
| **HIF-PHIs versus EPO or Placebo** | | | | | | | | | | | | | |
| HIF-PHIs | 2 | Takkavatakarn 2023^64^ | DD+NDD | 26 | 7844 | MD | 2.15 (-0.44, 4.75) | Random | 0.104 | 93.4 | <0.001 | High | Moderate |
| Roxadustat | 5 | Zheng 2021^71^ | DD+NDD | 5 | 1090 | MD | 7.07 (-1.13, 15.28) | Random | 0.09 | 77 | <0.0001 | Moderate | Moderate |
| **HIF-PHIs versus EPO** | | | | | | | | | | | | | |
| HIF-PHIs | 3 | Takkavatakarn 2023^64^ | DD+NDD | 24 | 4761 | MD | 4.81 (0.96, 8.67) | Random | <0.001 | 93.3 | 0.014 | High | Moderate |
| Roxadustat | 6 | Zheng 2023^72^ | DD | 8 | 3940 | MD | 3.25 (2.11, 4.38) | Random | <0.001 | 80 | <0.001 | Moderate | Low |
| Daprodustat | 3 | Fatima 2022^68^ | DD+NDD | 7 | 7707 | MD | 0.29 (-0.60, 1.192) | Random | 0.52 | 69 | 0.003 | High | Moderate |
| Molidustat | 2 | Zheng 2023^72^ | DD | 2 | 428 | MD | 1.35 (-0.71, 3.40) | Random | 0.2 | 79 | 0.03 | Low | Low |
| Enarodustat | 2 | Zheng 2023^72^ | DD | 1 | 192 | MD | 1.53 (-3.36, 6.42) | - | 0.54 | - |  | Low | Low |
| Desidustat | 2 | Zheng 2023^72^ | DD | 1 | 373 | MD | 0.15 (0.13, 0.17) | - | <0.001 | - |  | Low | Low |
| **HIF-PHIs versus Placebo** | | | | | | | | | | | | | |
| HIF-PHIs | 3 | Takkavatakarn 2023^64^ | DD+NDD | 20 | 3082 | MD | -1.44 (-5.61, 2.72) | Random | 0.497 | 78.4 | <0.001 | High | Moderate |
| Roxadustat | 5 | Zheng 2021^71^ | NDD | 3 | 328 | MD | -4.40 (-10.33, 1.53) | Random | 0.15 | 0 | 0.78 | Moderate | Moderate |
| Daprodustat | 1 | Li Jing 2021^73^ | NDD | 2 | 264 | MD | -7.75 (-15.23, -0.27) | Fixed | 0.04 | 0 | 0.93 | Low | Moderate |

Abbreviations: EPO, erythropoietin; DD, dialysis dependent; HIF-PHIs, hypoxia-inducible factor-prolyl hydroxylase inhibitors; MA, meta-analysis; MD, mean difference; NDD, non-dialysis dependent; SMD, standardized mean difference.

Supplementary Table 11. Efficacy of HIF-PHIs treatment on serum ferritin compared with erythropoietin replacement or placebo.

| **Experimental** | **Total eligible MA** | **Included MA** | **Targeted population** | **No. of primary studies** | **No. of patients** | **MA metrics** | **Estimates (95% CI)** | **Effects model** | **P-value** | **I^2^ (%)** | **P-value of Q test** | **NutriGrade** | **AMSTAR2** |
| --- | --- | --- | --- | --- | --- | --- | --- | --- | --- | --- | --- | --- | --- |
| **HIF-PHIs versus EPO or Placebo** | | | | | | | | | | | | | |
| HIF-PHIs | 3 | Takkavatakarn 2023^64^ | DD+NDD | 38 | 22568 | MD | -42.80 (-57.00, -28.59) | Random | <0.001 | 99.4 | <0.001 | High | Moderate |
| Roxadustat | 5 | Zheng 2021^71^ | DD+NDD | 8 | 2237 | MD | -38.35 (-67.41, -9.29) | Random | 0.01 | 80 | <0.00001 | High | Moderate |
| **HIF-PHIs versus EPO** | | | | | | | | | | | | | |
| HIF-PHIs | 3 | Takkavatakarn 2023^64^ | DD+NDD | 33 | 17072 | MD | -26.59 (-52.64, -0.54) | Random | 0.045 | 98.1 | <0.001 | High | Moderate |
| Roxadustat | 7 | Zheng 2023^72^ | DD | 9 | 4916 | SMD | -0.15 (-0.27, -0.03) | Random | 0.02 | 67 | 0.0007 | Moderate | Low |
| Daprodustat | 3 | Fatima 2022^68^ | DD+NDD | 8 | 7877 | MD | -17.29 (-29.69, -4.89) | Random | 0.006 | 40 | 0.12 | High | Moderate |
| Vadadustat | 2 | Zheng 2023^72^ | DD | 2 | 4246 | SMD | 0.06 (-0.19, 0.30) | Random | 0.66 | 86 | 0.0008 | Moderate | Low |
| Molidustat | 2 | Zheng 2023^72^ | DD | 2 | 428 | SMD | 0.33 (-0.26, 0.92) | Random | 0.27 | 86 | 0.008 | Low | Low |
| Enarodustat | 2 | Zheng 2023^72^ | DD | 1 | 172 | SMD | 0.13 (-0.17, 0.43) | - | 0.41 | - |  | Low | Low |
| Desidustat | 2 | Zheng 2023^72^ | DD | 1 | 392 | SMD | -0.05 (-0.25, 0.14) | - | 0.59 | - |  | Low | Low |
| **HIF-PHIs versus Placebo** | | | | | | | | | | | | | |
| HIF-PHIs | 3 | Takkavatakarn 2023^64^ | DD+NDD | 32 | 5496 | MD | -56.73 (-76.37, -37.09) | Random | <0.001 | 99.6 | <0.001 | High | Moderate |
| Roxadustat | 5 | Zheng 2021^71^ | NDD | 5 | 422 | MD | -51.91 (-61.75, -42.06) | Fixed | <0.00001 | 35 | 0.19 | Moderate | Moderate |
| Daprodustat | 2 | Li Jing 2021^73^ | NDD | 2 | 265 | MD | -30.15 (-50.89, -9.41) | Fixed | 0.004 | 25 | 0.26 | Low | Moderate |
| Vadadustat | 1 | Li Jing 2021^73^ | NDD | 2 | 297 | MD | -57.97 (-80.44, -35.50) | Fixed | <0.00001 | 0 | 0.91 | Low | Moderate |

Abbreviations: EPO, erythropoietin; DD, dialysis dependent; HIF-PHIs, hypoxia-inducible factor-prolyl hydroxylase inhibitors; MA, meta-analysis; MD, mean difference; NDD, non-dialysis dependent; SMD, standardized mean difference.

Supplementary Table 12. Efficacy of HIF-PHIs treatment on safety outcomes compared with erythropoietin replacement or placebo.

| **Experimental** | **Outcomes** | **Total eligible MAs** | **Included MA** | **Targeted population** | **No. of primary studies** | **No. of patients** | **MA metrics** | **Estimates (95% CI)** | **Effects model** | **P-value** | **I^2^ (%)** | **P-value of Q test** | **NutriGrade** | **AMSTAR2** |
| --- | --- | --- | --- | --- | --- | --- | --- | --- | --- | --- | --- | --- | --- | --- |
| **HIF-PHIs versus EPO or Placebo** | | | | | | | | | | | | | | |
| HIF-PHIs | Mortality | **1** | Takkavatakarn 2023^64^ | DD+NDD | 23 | 22113 | RR | 0.91 (0.78, 1.07) | Random | 0.245 | NR | NR | High | Moderate |
|  | MACE | 1 | Takkavatakarn 2023^64^ | DD+NDD | 12 | 16472 | RR | 1.00 (0.94, 1.07) | Random | 0.991 | NR | NR | High | Moderate |
|  | Stroke | 1 | Takkavatakarn 2023^64^ | DD+NDD | 19 | 19496 | RR | 0.97 (0.77, 1.23) | Random | 0.818 | NR | NR | High | Moderate |
| **HIF-PHIs versus EPO** | | | | | | | | | | | | | | |
| HIF-PHIs | Mortality | 1 | Mohamed 2023^66^ | NDD | 6 | 1236 | RR | 1.02 (0.92, 1.13) | Random | 0.71 | 0 | 0.88 | Low | Very low |
|  | AEs | 4 | Mohamed 2023^66^ | NDD | 7 | 8318 | RR | 1.01 (0.99, 1.01) | Random | 0.28 | 34 | 0.17 | Low | Very low |
|  | SAE | 4 | Takkavatakarn 2023^64^ | DD+NDD | 28 | 22602 | RR | 1.04 (0.99, 1.08) | Random | 0.53 | NR | NR | High | Moderate |
|  | MACE | 4 | Mohamed 2023^66^ | NDD | 4 | 1651 | RR | 1.08 (0.99, 1.18) | Random | 0.08 | 0 | 0.62 | Low | Very low |
| Roxadustat | Mortality | 1 | Qie 2021^74^ | DD+NDD | 3 | 544 | RR | 2.38 (0.13, 44.93) | Random | 0.56 | NR | NR | Moderate | Low |
|  | AEs | 5 | Liu 2021^75^ | DD+NDD | 4 | 846 | RR | 1.22 (0.91, 1.64) | Random | 0.18 | 60 | 0.06 | High | Moderate |
|  | SAE | 2 | Zheng 2021^71^ | DD+NDD | 5 | 1680 | OR | 1.33 (1.06, 1.68) | Fixed | 0.01 | 0 | 0.9 | High | Moderate |
|  | MACE | 1 | Qie 2021^74^ | DD+NDD | 2 | 448 | RR | 1.30 (0.22, 7.62) | Fixed | NR | NR | NR | Moderate | Low |
|  | Stroke | 1 | Qie 2021^74^ | DD+NDD | 1 | 144 | RR | 1.70 (0.08, 34.55) | Random | NR | NR | NR | Low | Low |
| Daprodustat | Mortality | 1 | Fatima 2022^68^ | DD+NDD | 5 | NR | RR | 1.00 (0.90, 1.11） | Random | NR | 0 | 1 | Moderate | Moderate |
|  | MACE | 1 | Fatima 2022^68^ | DD+NDD | 5 | 7614 | RR | 0.98 (0.84, 1.14) | Random | 0.8 | 45 | 0.12 | High | Moderate |
|  | Stroke | 1 | Fatima 2022^68^ | DD+NDD | 5 | NR | RR | 1.01 (0.61, 1.67) | Random | 0.96 | 42 | NR | Moderate | Moderate |
| **HIF-PHIs versus Placebo** | | | | | | | | | | | | | | |
| HIF-PHIs | AEs | 3 | Zhang 2021^76^ | NDD | 8 | 989 | RR | 0.98 (0.88, 1.10) | Random | 0.74 | 10 | 0.35 | High | Low |
|  | SAE | 1 | Wu 2022^77^ | DD | 11 | 438 | RR | 0.90 (0.45, 1.81) | Random | 0.78 | 0 | 0.98 | Moderate | Moderate |
|  | MACE | 1 | Wang 2020^70^ | DD+NDD | 6 | NR | RR | 1.48 (0.47, 4.71) | Random | NR | 0 | 0.61 | Moderate | Low |
| Roxadustat | AEs | 5 | Liu 2021^75^ | DD+NDD | 7 | 4736 | RR | 1.02 (1.00, 1.04) | Random | 0.08 | 0 | 0.54 | High | Moderate |
|  | SAE | 3 | Zheng 2021^71^ | DD+NDD | 5 | 1060 | OR | 1.19 (0.88, 1.62) | Fixed | 0.26 | 0 | 0.86 | High | Moderate |
|  | MACE | 2 | Qie 2021^74^ | DD+NDD | 2 | 223 | RR | 0.55 (0.07, 4.11) | Random | NR | NR | NR | Low | Low |

Abbreviations: AE, adverse events; EPO, erythropoietin; DD, dialysis dependent; HIF-PHIs, hypoxia-inducible factor-prolyl hydroxylase inhibitors; MA, meta-analysis; MACE: major adverse cardiovascular events; MD, mean difference; NDD, non-dialysis dependent; NR, not reported; OR: odds ratio; RR: risk ratio; SAE, severe adverse events SMD, standardized mean difference.

Supplementary Figure 1. Efficacy of HIF-PHIs treatment on serum iron and ferritin compared with erythropoietin replacement or placebo.


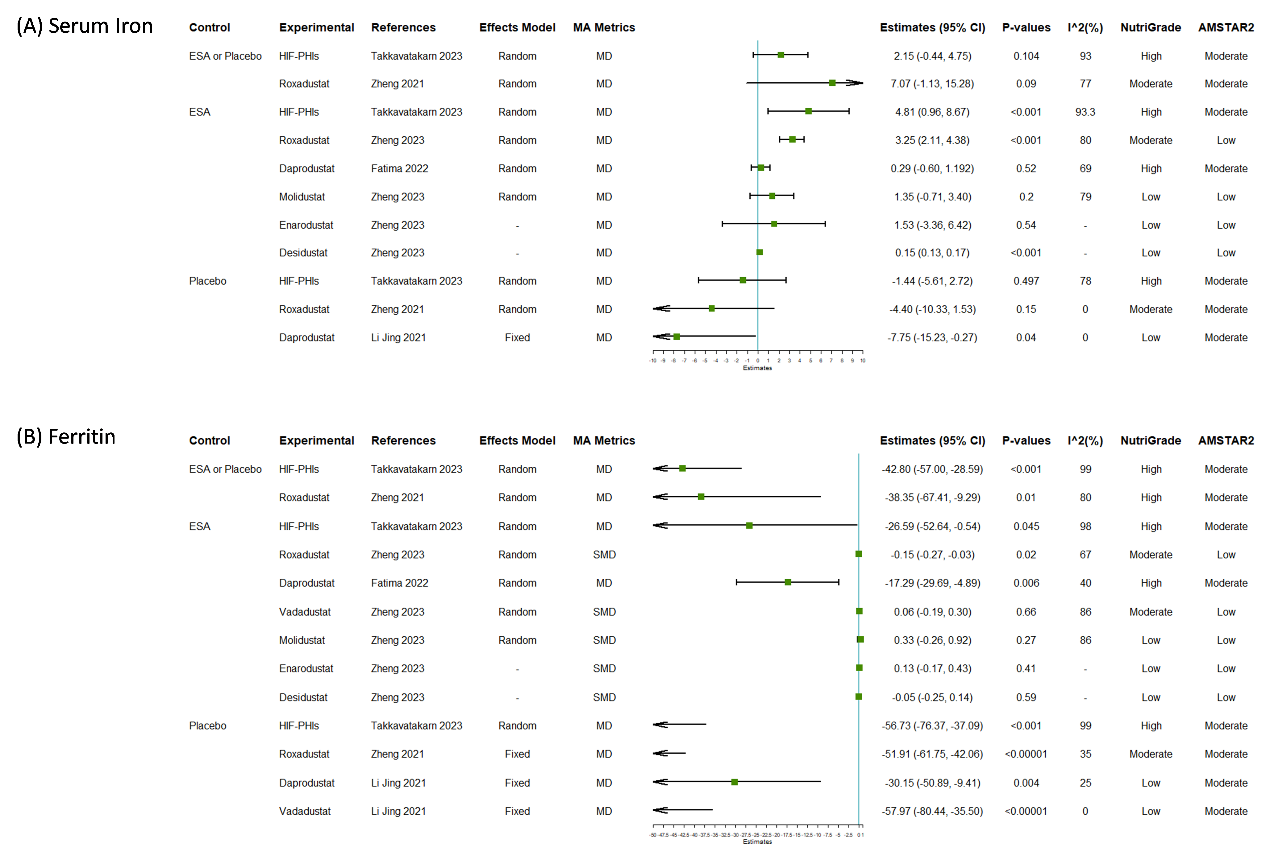


Abbreviations: EPO, erythropoietin; HIF-PHIs, hypoxia-inducible factor-prolyl hydroxylase inhibitors; MD, mean differences; NR, not reported; SMD, standardized mean differences.

References

1. Farag YMK, Zheng Z, Luo W. Letter to the Editor in response to the article "Efficacy and safety of HIF prolyl-hydroxylase inhibitor vs epoetin and darbepoetin for anemia in chronic kidney disease patients not undergoing dialysis: a network meta-analysis". *Pharmacological research*. 2020:105213. doi:https://dx.doi.org/10.1016/j.phrs.2020.105213

2. Zheng Q, Yang H, Liu YN, Liu WJ. In response to "Title: Letter to the Editor in response to the article 'Efficacy and safety of HIF prolyl hydroxylase inhibitor vs epoetin and darbepoetin for anemia in chronic kidney disease patients not undergoing dialysis: A network meta-analysis'". *Pharmacological Research*. 2021;163:105227. doi:https://dx.doi.org/10.1016/j.phrs.2020.105227

3. Atzinger C, Cichewicz A, Huelin R, Alexandre AF. Efficacy and Safety of Hypoxia-Inducible Factor Prolyl Hydroxylase Inhibitors for Treatment of Anaemia of Chronic Kidney Disease: A Systematic Literature Review. *Value in Health*. 2022;25(12 Supplement):S25. ISPOR Europe 2022. Vienna Austria. doi:https://dx.doi.org/10.1016/j.jval.2022.09.121

4. Fukuta H, Hagiwara H, Kamiya T. Hypoxia-inducible factor prolyl hydroxylase inhibitors for anemia in heart failure patients: A protocol for systematic review and meta-analysis. *PLoS ONE*. 2022;17(9 September):e0275311. doi:https://dx.doi.org/10.1371/journal.pone.0275311

5. Natale P, Palmer SC, Tong A, et al. Hypoxia-inducible factor stabilisers for the anaemia of chronic kidney disease. *Cochrane Database of Systematic Reviews*. 2020;2020(10):CD013751. doi:https://dx.doi.org/10.1002/14651858.CD013751

6. Alshamsi I. Extended Literature Review of the role of erythropoietin stimulating agents (ESA) use in the management of post renal transplant anaemia. *Transplantation Reports*. 2022;7(2):100097. doi:https://dx.doi.org/10.1016/j.tpr.2022.100097

7. Deicher R, Horl WH. Anaemia as a risk factor for the progression of chronic kidney disease. *Current Opinion in Nephrology and Hypertension*. 2003;12(2):139-143. doi:https://dx.doi.org/10.1097/00041552-200303000-00003

8. Del Vecchio L, Locatelli F. An overview on safety issues related to erythropoiesis-stimulating agents for the treatment of anaemia in patients with chronic kidney disease. *Expert Opinion on Drug Safety*. 2016;15(8):1021-1030. doi:https://dx.doi.org/10.1080/14740338.2016.1182494

9. Harlow CE, Gandawijaya J, Bamford RA, et al. Identification and single-base gene-editing functional validation of a cis-EPO variant as a genetic predictor for EPO-increasing therapies. *American Journal of Human Genetics*. 2022;109(9):1638-1652. doi:https://dx.doi.org/10.1016/j.ajhg.2022.08.004

10. Macdougall IC. Iron therapy for managing anaemia in chronic kidney disease. *Curr Opin Nephrol Hypertens*. Sep 2018;27(5):358-363. doi:10.1097/mnh.0000000000000436

11. Schiller B, Besarab A. Simplifying anemia management in hemodialysis patients: ESAs administered at longer dosing intervals can enhance opportunities to provide patient-focused care. *Current Medical Research and Opinion*. 2011;27(8):1539-1550. doi:https://dx.doi.org/10.1185/03007995.2011.588202

12. Zhang HY, Cheng M, Zhang L, Wang YP. Ferroptosis and renal fibrosis: A new target for the future (Review). *Experimental and Therapeutic Medicine*. 2023;25(1):13. doi:https://dx.doi.org/10.3892/etm.2022.11712

13. Sasongko TH, Ismail NFD, Zabidi‐Hussin Z. Rapamycin and rapalogs for tuberous sclerosis complex. *Cochrane Database of Systematic Reviews*. 2016;(7)doi:10.1002/14651858.CD011272.pub2

14. Coronado Daza J, Martí‐Carvajal AJ, Ariza García A, et al. Early versus delayed erythropoietin for the anaemia of end‐stage kidney disease. *Cochrane Database of Systematic Reviews*. 2015;(12)doi:10.1002/14651858.CD011122.pub2

15. Tonia T, Mettler A, Robert N, et al. Erythropoietin or darbepoetin for patients with cancer. *Cochrane Database of Systematic Reviews*. 2012;(12)doi:10.1002/14651858.CD003407.pub5

16. Nieto Estrada VH, Molano Franco D, Medina RD, Gonzalez Garay AG, Martí‐Carvajal AJ, Arevalo‐Rodriguez I. Interventions for preventing high altitude illness: Part 1. Commonly‐used classes of drugs. *Cochrane Database of Systematic Reviews*. 2017;(6)doi:10.1002/14651858.CD009761.pub2

17. Carson JL, Stanworth SJ, Dennis JA, et al. Transfusion thresholds for guiding red blood cell transfusion. *Cochrane Database of Systematic Reviews*. 2021;(12)doi:10.1002/14651858.CD002042.pub5

18. Fishbane S, Provenzano R, Pergola P, et al. POS-258 CARDIOVASCULAR OUTCOMES AND EXPLORATORY ANALYSES BY ACHIEVED HB LEVELS IN THE POOLED PHASE 3 ROXADUSTAT STUDIES OF NON-DIALYSIS-DEPENDENT PATIENTS WITH ANEMIA OF CHRONIC KIDNEY DISEASE. *Kidney International Reports*. 2021;6(4 Supplement):S109-S110. ISN World Congress of Nephrolog. Montreal Canada. doi:https://dx.doi.org/10.1016/j.ekir.2021.03.273

19. Pollock C, Roger S, Manllo-Karim R, et al. POS-256 ROXADUSTAT INCREASES HEMOGLOBIN IN ANEMIC NON-DIALYSIS-DEPENDENT (NDD) AND DIALYSIS-DEPENDENT (DD) CHRONIC KIDNEY DISEASE (CKD) PATIENTS INDEPENDENT OF INFLAMMATION. *Kidney International Reports*. 2021;6(4 Supplement):S108. ISN World Congress of Nephrolog. Montreal Canada. doi:https://dx.doi.org/10.1016/j.ekir.2021.03.271

20. Provenzano R, Fishbane S, Pergola P, et al. POS-284 CARDIOVASCULAR OUTCOMES AND EXPLORATORY ANALYSES BY ACHIEVED HB LEVELS IN POOLED PHASE 3 TRIALS OF ROXADUSTAT IN DIALYSIS-DEPENDENT PATIENTS WITH ANEMIA OF CHRONIC KIDNEY DISEASE. *Kidney International Reports*. 2021;6(4 Supplement):S121. ISN World Congress of Nephrolog. Montreal Canada. doi:https://dx.doi.org/10.1016/j.ekir.2021.03.299

21. Putra BP, Putra FN. POS-285 EXPANDING THE POTENTIAL BENEFIT OF HYPOXIA-INDUCIBLE FACTOR PROLYL HYDROXYLASE INHIBITORS FOR IMPROVING DYSLIPIDEMIA IN ANEMIA OF CHRONIC KIDNEY DISEASE PATIENTS: META-ANALYSIS OF RANDOMIZED CONTROLLED TRIALS. *Kidney International Reports*. 2021;6(4 Supplement):S121-S122. ISN World Congress of Nephrolog. Montreal Canada. doi:https://dx.doi.org/10.1016/j.ekir.2021.03.300

22. Yang J, Zhang X, Wang L. POS-354 EFFECTS OF HYPOXIA-INDUCIBLE FACTOR PROLYL HYDROXYLASE INHIBITORS VS ERYTHROPOIESIS-STIMULATING AGENTS ON IRON REGULATION IN NON-DIALYSIS-DEPENDENT ANEMIC PATIENTS WITH CKD: A NETWORK META-ANALYSIS. *Kidney International Reports*. 2022;7(2 Supplement):S159-S160. ISN World Congress of Nephrology (WCN). Kuala Lumpur Malaysia. doi:https://dx.doi.org/10.1016/j.ekir.2022.01.375

23. Natale P, Palmer S, Tong A, et al. Hypoxia-inducible factor stabilisers for the anaemia of chronic kidney disease: A Cochrane review. *Nephrology*. 2021;26(SUPPL 2):48-49. 56th Annual Scientific Meeting of the Australian and New Zealand Society of Nephrology, ANZSN 2021. Virtual. doi:https://dx.doi.org/10.1111/nep.13932

24. Borawski B, Malyszko JS, Kwiatkowska M, Malyszko J. Current status of renal anemia pharmacotherapy-what can we offer today. *Journal of Clinical Medicine*. 2021;10(18):4149. doi:https://dx.doi.org/10.3390/jcm10184149

25. Del Vecchio L, Locatelli F. Investigational hypoxia-inducible factor prolyl hydroxylase inhibitors (HIF-PHI) for the treatment of anemia associated with chronic kidney disease. *Expert Opinion on Investigational Drugs*. 2018;27(7):613-621. doi:https://dx.doi.org/10.1080/13543784.2018.1493455

26. Faivre A, Scholz CC, de Seigneux S. Hypoxia in chronic kidney disease: towards a paradigm shift? *Nephrology, dialysis, transplantation : official publication of the European Dialysis and Transplant Association - European Renal Association*. 2020;doi:https://dx.doi.org/10.1093/ndt/gfaa091

27. Li J, Haase VH, Hao CM. Updates on Hypoxia-Inducible Factor Prolyl Hydroxylase Inhibitors in the Treatment of Renal Anemia. *Kidney Diseases*. 2023;9(1)doi:https://dx.doi.org/10.1159/000527835

28. Locatelli F, Minutolo R, De Nicola L, Del Vecchio L. Evolving Strategies in the Treatment of Anaemia in Chronic Kidney Disease: The HIF-Prolyl Hydroxylase Inhibitors. *Drugs*. 2022;82(16):1565-1589. doi:https://dx.doi.org/10.1007/s40265-022-01783-3

29. López-Gómez JM, Abad S, Vega A. New expectations in the treatment of anemia in chronic kidney disease. *Nefrologia : publicacion oficial de la Sociedad Espanola Nefrologia*. May-Jun 2016;36(3):232-6. Nuevas expectativas en el tratamiento de la anemia en la enfermedad renal crónica. doi:10.1016/j.nefro.2016.03.006

30. Magwood JS, Lebby A, Chen B, Kessler S, Norris L, Bennett CL. Emerging drugs for treatment of anemia of chronic kidney disease. *Expert Opinion on Emerging Drugs*. 2013;18(4):421-429. doi:https://dx.doi.org/10.1517/14728214.2013.836490

31. Musio F. Revisiting the treatment of anemia in the setting of chronic kidney disease, hematologic malignancies, and cancer: perspectives with opinion and commentary. *Expert Review of Hematology*. 2020;13(11):1175-1188. doi:https://dx.doi.org/10.1080/17474086.2020.1830371

32. Schmid H, Schiffl H, Lederer SR. New strategies for managing anemia of chronic kidney disease. *Cardiovascular and Hematological Agents in Medicinal Chemistry*. 2012;10(4):339-351. doi:https://dx.doi.org/10.2174/187152512803530342

33. Souza E, Cho KH, Harris ST, Flindt NR, Watt RK, Pai AB. Hypoxia-inducible factor prolyl hydroxylase inhibitors: a paradigm shift for treatment of anemia in chronic kidney disease? *Expert Opinion on Investigational Drugs*. 2020;29(8):831-844. doi:https://dx.doi.org/10.1080/13543784.2020.1777276

34. Visweswaran V, Pavithran K. Belzutifan: A Narrative Drug Review. *Current Drug Research Reviews*. 2022;14(2):88-95. doi:https://dx.doi.org/10.2174/2589977514666220401094724

35. Zhu SM, Yang HY, Wang JT. Research advances in hypoxia-inducible factor prolyl hydroxylase inhibitors for the treatment of renal anemia. *Chinese Journal of New Drugs*. 2017;26(22):2701-2705.

36. Kan C, Lu X, Zhang R. Effects of hypoxia on bone metabolism and anemia in patients with chronic kidney disease. *World Journal of Clinical Cases*. 2021;9(34):10616-10625. doi:https://dx.doi.org/10.12998/wjcc.v9.i34.10616

37. Mokiou S, Hakimi Z, Wang-Silvanto J, Hollier-Hann G, Moeller A. USE OF HEALTH-RELATED QUALITY OF LIFE (HRQOL) INSTRUMENTS IN CLINICAL TRIALS OF ANAEMIA OF CHRONIC KIDNEY DISEASE (CKD). *Value in Health*. 2018;21(Supplement 3):S481. ISPOR Europe 2018: New Perspectives for Improving 21st Century Health Systems. Barcelona Spain. doi:https://dx.doi.org/10.1016/j.jval.2018.09.2835

38. Zheng L, Liu M, Zhang Y, Zhang K, Gu Y, Liu D. Bibliometric analysis of hypoxia inducible factor prolyl hydroxylase inhibitor in anemia. *Frontiers in Pharmacology*. 2022;13:1005225. doi:https://dx.doi.org/10.3389/fphar.2022.1005225

39. Chen J, Shou X, Xu Y, et al. A network meta-analysis of the efficacy of hypoxia-inducible factor prolyl-hydroxylase inhibitors in dialysis chronic kidney disease. *Aging*. Mar 27 2023;15(6):2237-2274. doi:10.18632/aging.204611

40. Fadlalmola H, Al-Sayaghi K, Al-Hebshi A, et al. Efficacy of Different Doses of Daprodustat for Anemic Non-dialysis Patients with Chronic Kidney Disease: A Systematic Review and Network Meta-Analysis. *Journal of Clinical Medicine*. 2022;11(10):2722. doi:https://dx.doi.org/10.3390/jcm11102722

41. Yang J, Xing J, Zhu X, Xie X, Wang L, Zhang X. Effects of hypoxia-inducible factor-prolyl hydroxylase inhibitors vs. erythropoiesis-stimulating agents on iron metabolism in non-dialysis-dependent anemic patients with CKD: A network meta-analysis. *Frontiers in Endocrinology*. 2023;14:1131516. doi:https://dx.doi.org/10.3389/fendo.2023.1131516

42. Zheng Q, Yang H, Sun L, et al. Efficacy and safety of HIF prolyl-hydroxylase inhibitor vs epoetin and darbepoetin for anemia in chronic kidney disease patients not undergoing dialysis: a network meta-analysis. *Pharmacological research*. 2020:105020. doi:https://dx.doi.org/10.1016/j.phrs.2020.105020

43. Chung EYM, Palmer SC, Saglimbene VM, Craig JC, Tonelli M, Strippoli GFM. Erythropoiesis‐stimulating agents for anaemia in adults with chronic kidney disease: a network meta‐analysis. *Cochrane Database of Systematic Reviews*. 2023;(2)doi:10.1002/14651858.CD010590.pub3

44. Barratt J, Dimkovic N, Shutov E, et al. Pooled efficacy and cardiovascular safety results of 3 placebo-controlled and 1 darbepoetin alfa-controlled studies of roxadustat for treatment of anaemia in patients with non-dialysis-dependent chronic kidney disease. *Nephrology Dialysis Transplantation*. 2021;36(SUPPL 1):i51. 58th ERA-EDTA Congress. Virtual. doi:https://dx.doi.org/10.1093/ndt/gfab118.003

45. Barratt J, Sulowicz W, Cockburn E, Reusch M, Young J, Dimkovic N. Cardiovascular outcomes associated with achieved haemoglobin level in pooled phase 3 studies of roxadustat in non-dialysis-dependent patients with anaemia. *Nephrology Dialysis Transplantation*. 2021;36(SUPPL 1):i326. 58th ERA-EDTA Congress. Virtual. doi:https://dx.doi.org/10.1093/ndt/gfab085.003

46. Chan TMD, Pecoits-Filho R, Rastogi A, et al. Roxadustat vs. Placebo or epoetin alfa has no clinically meaningful effect on blood pressure in patients with anemia of CKD. *Journal of the American Society of Nephrology*. 2020;31:649. Kidney Week 2020. Virtual United States.

47. Fishbane S, Provenzano R, Pergola PE, et al. Associations between achieved hemoglobin and cardiovascular outcomes in the pooled phase 3 roxadustat studies of non-dialysis-dependent patients with anemia of CKD. *Journal of the American Society of Nephrology*. 2020;31:B4. Kidney Week 2020. Virtual United States.

48. Parfrey PS, Luo W, Maroni B, Anders R, Vargo D, McCullough PA. Thromboembolic events with vadadustat vs. darbepoetin alfa for anemia treatment in patients with dialysis-dependent CKD. *Journal of the American Society of Nephrology*. 2021;32:184. Kidney Week 2021. San Diego, CA United States.

49. Provenzano R, Fishbane S, Pergola PE, et al. Associations between achieved hemoglobin and cardiovascular outcomes in the pooled phase 3 trials of roxadustat in dialysis-dependent patients with anemia of CKD. *Journal of the American Society of Nephrology*. 2020;31:B4-B5. Kidney Week 2020. Virtual United States.

50. McCullough PA, Luo W, Anders R, Vargo D, Parfrey PS. Assessment of thromboembolic events with vadadustat vs. darbepoetin alfa for treatment of anemia in patients with non-dialysis-dependent CKD. *Journal of the American Society of Nephrology*. 2021;32:184. Kidney Week 2021. San Diego, CA United States.

51. Patoulias D, Papadopoulos C, Doumas M. Meta-Analysis Addressing the Cardiovascular Safety of Daprodustat in Patients With Chronic Kidney Disease Undergoing Dialysis or Not. *American Journal of Cardiology*. 2022;170:166-167. doi:https://dx.doi.org/10.1016/j.amjcard.2022.02.003

52. Hahn D, Esezobor CI, Elserafy N, Webster AC, Hodson EM. Short‐acting erythropoiesis‐stimulating agents for anaemia in predialysis patients. *Cochrane Database of Systematic Reviews*. 2017;(1)doi:10.1002/14651858.CD011690.pub2

53. Chen H, Cheng Q, Wang J, Zhao X, Zhu S. Long-term efficacy and safety of hypoxia-inducible factor prolyl hydroxylase inhibitors in anaemia of chronic kidney disease: A meta-analysis including 13,146 patients. *Journal of Clinical Pharmacy and Therapeutics*. 2021;46(4):999-1009. doi:https://dx.doi.org/10.1111/jcpt.13385

54. Fu Z, Geng X, Chi K, et al. Efficacy and Safety of Daprodustat Vs rhEPO for Anemia in Patients With Chronic Kidney Disease: A Meta-Analysis and Trial Sequential Analysis. *Frontiers in Pharmacology*. 2022;13:746265. doi:https://dx.doi.org/10.3389/fphar.2022.746265

55. Jia L, Dong X, Yang J, Jia R, Zhang H. Effectiveness of hypoxia-inducible factor prolyl hydroxylase inhibitor roxadustat on renal anemia in non-dialysis-dependent chronic kidney disease: A systematic review and meta-analysis. *Annals of Translational Medicine*. 2019;7(23):09. doi:https://dx.doi.org/10.21037/atm.2019.12.18

56. Khor SY, Alattal S, Kamboj AS, et al. Efficacy and Safety of Daprodustat for Treatment of Anemia in CKD: A Meta-Analysis. *Journal of the American Society of Nephrology*. 2022;33:243. Kidney Week 2022. Orlando, FL United States.

57. Li M, Lan J, Dong F, Duan P. Effectiveness of hypoxia-induced factor prolyl hydroxylase inhibitor for managing anemia in chronic kidney disease: a systematic review and meta-analysis. *European Journal of Clinical Pharmacology*. 2021;77(4):491-507. doi:https://dx.doi.org/10.1007/s00228-020-03037-1

58. Natale P, Palmer SC, Jaure A, et al. Hypoxia-inducible factor stabilisers for the anaemia of chronic kidney disease. *Cochrane Database of Systematic Reviews*. 2022;2022(8):CD013751. doi:https://dx.doi.org/10.1002/14651858.CD013751.pub2

59. Wang L, Yin H, Yang L, Zhang F, Wang S, Liao D. The Efficacy and Safety of Roxadustat for Anemia in Patients With Chronic Kidney Disease: A Meta-Analysis. *Frontiers in Pharmacology*. 2022;13:779694. doi:https://dx.doi.org/10.3389/fphar.2022.779694

60. Wen T, Zhang X, Wang Z, Zhou R. Hypoxia-Inducible Factor Prolyl Hydroxylase Inhibitors in Patients with Renal Anemia: A Meta-Analysis of Randomized Trials. *Nephron*. 2020:1-11. doi:https://dx.doi.org/10.1159/000508812

61. Xie D, Wang J, Wu X, Li M. Effect of daprodustat on anemia in patients with chronic kidney disease: a meta-analysis. *Int Urol Nephrol*. Dec 2018;50(12):2201-2206. doi:10.1007/s11255-018-1940-8

62. Zheng Q, Wang Y, Yang H, et al. Cardiac and Kidney Adverse Effects of HIF Prolyl-Hydroxylase Inhibitors for Anemia in Patients With CKD Not Receiving Dialysis: A Systematic Review and Meta-analysis. *American Journal of Kidney Diseases*. 2023;81(4):434-445.e1. doi:https://dx.doi.org/10.1053/j.ajkd.2022.09.014

63. Zhong H, Zhou T, Li H, Zhong Z. The role of hypoxia-inducible factor stabilizers in the treatment of anemia in patients with chronic kidney disease. *Drug Design, Development and Therapy*. 2018;12:3003-3011. doi:https://dx.doi.org/10.2147/DDDT.S175887

64. Takkavatakarn K, Thammathiwat T, Phannajit J, et al. The impacts of hypoxia-inducible factor stabilizers on laboratory parameters and clinical outcomes in chronic kidney disease patients with renal anemia: a systematic review and meta-analysis. *Clinical kidney journal*. May 2023;16(5):845-858. doi:10.1093/ckj/sfac271

65. Liu J, Zhang A, Hayden JC, et al. Roxadustat (FG-4592) treatment for anemia in dialysis-dependent (DD) and not dialysis-dependent (NDD) chronic kidney disease patients: A systematic review and meta-analysis. *Pharmacological Research*. 2020;155:104747. doi:https://dx.doi.org/10.1016/j.phrs.2020.104747

66. Mohamed MMG, Oyenuga M, Shaikh S, Oyenuga A, Kheiri B, Nwankwo C. Hypoxia inducible factor-prolyl hydroxylase inhibitors in anemic patients with non-dialysis dependent chronic kidney disease: a meta-analysis of randomized clinical trials. *International Urology and Nephrology*. 2023;55(1):167-171. doi:https://dx.doi.org/10.1007/s11255-022-03300-7

67. Lei J, Li H, Wang S. Efficacy and Safety of Roxadustat in Patients with Chronic Kidney Disease: An Updated Meta-Analysis of Randomized Controlled Trials including 6,518 Patients. *BioMed Research International*. 2022;2022:2413176. doi:https://dx.doi.org/10.1155/2022/2413176

68. Fatima K, Ahmed W, Fatimi AS, et al. Evaluating the safety and efficacy of daprodustat for anemia of chronic kidney disease: a meta-analysis of randomized clinical trials. *European Journal of Clinical Pharmacology*. 2022;78(12):1867-1875. doi:https://dx.doi.org/10.1007/s00228-022-03395-y

69. Xiong L, Zhang H, Guo Y, Song Y, Tao Y. Efficacy and Safety of Vadadustat for Anemia in Patients With Chronic Kidney Disease: A Systematic Review and Meta-Analysis. *Frontiers in Pharmacology*. 2021;12:795214. doi:https://dx.doi.org/10.3389/fphar.2021.795214

70. Wang B, Yin Q, Han YC, et al. Effect of hypoxia-inducible factor-prolyl hydroxylase inhibitors on anemia in patients with CKD: a meta-analysis of randomized controlled trials including 2804 patients. *Renal Failure*. 2020;42(1):912-925. doi:https://dx.doi.org/10.1080/0886022X.2020.1811121

71. Zheng L, Tian J, Liu D, et al. Efficacy and safety of roxadustat for anaemia in dialysis-dependent and non-dialysis-dependent chronic kidney disease patients: a systematic review and meta-analysis. *British journal of clinical pharmacology*. 2021;doi:https://dx.doi.org/10.1111/bcp.15055

72. Zheng Q, Zhang P, Yang H, et al. Effects of hypoxia-inducible factor prolyl hydroxylase inhibitors versus erythropoiesis-stimulating agents on iron metabolism and inflammation in patients undergoing dialysis: A systematic review and meta-analysis. *Heliyon*. Apr 2023;9(4):e15310. doi:10.1016/j.heliyon.2023.e15310

73. Li J, Xie QH, You L, Xu NX, Hao CM. Effects of hypoxia-inducible factor prolyl hydroxylase inhibitors on iron regulation in non-dialysis-dependent chronic kidney disease patients with anemia: A systematic review and meta-analysis. *Pharmacological Research*. 2021;163:105256. doi:https://dx.doi.org/10.1016/j.phrs.2020.105256

74. Qie S, Jiao N, Duan K, Li J, Liu Y, Liu G. The efficacy and safety of roxadustat treatment for anemia in patients with kidney disease: a meta-analysis and systematic review. *International Urology and Nephrology*. 2021;53(5):985-997. doi:https://dx.doi.org/10.1007/s11255-020-02693-7

75. Liu C, Fu Z, Jiang J, et al. Safety and Efficacy of Roxadustat for Anemia in Patients With Chronic Kidney Disease: A Meta-Analysis and Trial Sequential Analysis. *Frontiers in Medicine*. 2021;8:724456. doi:https://dx.doi.org/10.3389/fmed.2021.724456

76. Zhang S, Guo J, Xie S, Chen J, Yu S, Yu Y. Efficacy and safety of hypoxia-inducible factor prolyl hydroxylase inhibitor (HIF-PHI) on anemia in non-dialysis-dependent chronic kidney disease (NDD-CKD): a systematic review and meta-analysis. *International Urology and Nephrology*. 2021;53(6):1139-1147. doi:https://dx.doi.org/10.1007/s11255-020-02671-z

77. Wu M, Zang C, Ma F, Chen B, Liu J, Xu Z. Hypoxia-inducible factor prolyl hydroxylase inhibitors for anaemia in maintenance dialysis: a meta-analysis. *Clinical and Experimental Nephrology*. 2022;26(11):1043-1054. doi:https://dx.doi.org/10.1007/s10157-022-02263-4
